# Development of a chemically defined medium for *Paenibacillus polymyxa* by parallel online monitoring of the respiration activity in microtiter plates

**DOI:** 10.1186/s12896-023-00793-7

**Published:** 2023-07-28

**Authors:** Jennifer Goldmanns, Georg Andreas Röhling, Marie Kristine Lipa, Theresa Scholand, Alexander Deitert, Tobias May, Evangeline Priya Haas, Matthias Boy, Andrea Herold, Jochen Büchs

**Affiliations:** 1 RWTH Aachen University, AVT – Biochemical Engineering, Forckenbeckstraße 51, 52074 Aachen, Germany; 2 BASF SE, Carl-Bosch-Straße 38, Ludwigshafen am Rhein, 67056 Germany

**Keywords:** *Paenibacillus polymyxa*, Chemically defined media, Online monitoring, Medium optimization, Growth limitations

## Abstract

**Background:**

One critical parameter in microbial cultivations is the composition of the cultivation medium. Nowadays, the application of chemically defined media increases, due to a more defined and reproducible fermentation performance than in complex media. In order, to improve cost-effectiveness of fermentation processes using chemically defined media, the media should not contain nutrients in large excess. Additionally, to obtain high product yields, the nutrient concentrations should not be limiting. Therefore, efficient medium optimization techniques are required which adapt medium compositions to the specific nutrient requirements of microorganisms.

**Results:**

Since most *Paenibacillus* cultivation protocols so far described in literature are based on complex ingredients, in this study, a chemically defined medium for an industrially relevant *Paenibacillus polymyxa* strain was developed. A recently reported method, which combines a systematic experimental procedure in combination with online monitoring of the respiration activity, was applied and extended to identify growth limitations for *Paenibacillus polymyxa*. All cultivations were performed in microtiter plates. By systematically increasing the concentrations of different nutrient groups, nicotinic acid was identified as a growth-limiting component. Additionally, an insufficient buffer capacity was observed. After optimizing the growth in the chemically defined medium, the medium components were systematically reduced to contain only nutrients relevant for growth. Vitamins were reduced to nicotinic acid and biotin, and amino acids to methionine, histidine, proline, arginine, and glutamate. Nucleobases/-sides could be completely left out of the medium. Finally, the cultivation in the reduced medium was reproduced in a laboratory fermenter.

**Conclusion:**

In this study, a reliable and time-efficient high-throughput methodology was extended to investigate limitations in chemically defined media. The interpretation of online measured respiration activities agreed well with the growth performance of samples measured in parallel via offline analyses. Furthermore, the cultivation in microtiter plates was validated in a laboratory fermenter. The results underline the benefits of online monitoring of the respiration activity already in the early stages of process development, to avoid limitations of medium components, oxygen limitation and pH inhibition during the scale-up.

**Supplementary Information:**

The online version contains supplementary material available at 10.1186/s12896-023-00793-7.

## Background

Finding suitable process parameters for growth of microorganisms and product formation in microbial cultivations is essential to achieve high product yields. One crucial parameter is the composition of the cultivation medium [1–3]. Often, media containing complex nutrient sources such as yeast extract, meat extract, peptone, and soy flour are applied [4] due to several reasons: It is well known that adding complex components to cultivations leads to high productivities [5]. Furthermore, media recipes are easy-to-use, due to the nutrient-rich composition of complex components. For example, yeast extract contains many nutrients such as carbohydrates, amino acids, peptides, vitamins, growth factors, and trace elements [4]. In contrast to those advantages, the varying composition of complex components often results in an inconsistent fermentation performance [6, 7]. Klotz et al. showed the impact of different compositions of yeast extract and peptone from various manufacturers on lactic acid concentrations and productivity of *Sporolactobacillus inulinus* [7]. Furthermore, the composition of soy flour and soy meal is influenced by the soybeans’ origin, climate and soil [8] and by their processing methods [9, 10]. Additionally, varying compositions of soy flour were shown over eight years in a single manufacturing plant [11].

To overcome these disadvantages of complex media, the application of chemically defined media is favorable. Chemically defined media are already used in various investigations, such as in studies of recombinant protein production [12], cell morphology [13], and metabolic engineering [14]. The advantages of chemically defined media were summarized by Zhang and Greasham [15]. As an example, these media can ensure a more reproducible fermentation performance explicitly due to the chemically defined composition. Furthermore, the media components usually are less sensitive to sterilization conditions, in contrast to complex components. Additionally, downstream processing is often simplified, as those media typically do not contain insoluble components, proteins or other cellular compounds [15]. However, cell densities and product yields are often lower in chemically defined media than in complex media [16, 17]. In addition, growth rates are often reduced, compared to complex media [17, 18]. Microorganisms need to synthesize various cellular building blocks on their own, when they are not supplied in the medium [15]. Growth in a chemically defined medium is not possible at all, if microorganisms exhibit auxotrophies and the required nutrient is not added to the medium [19]. In contrast to complex media, which can balance nutritional limitations, a significant challenge of applying a chemically defined medium is to identify the nutrients that the organisms need for high growth rates and product formation [15]. Nutrient requirements and necessary concentrations of nutrients need to be determined to achieve comparable or higher growth rates, cell densities and product yields than in complex media [16, 17, 20]. Additionally, adding unnecessary medium components or components in excess increases costs. As the specific demand for nutrients of microorganisms or strains varies, time-intensive medium optimization is necessary.

To identify and optimize suitable cultivation media, various techniques are established and were summarized by Singh et al. [21]. One classical method is the one-factor-at-a-time (OFAT) method. The technique is known to be easy to use, as only one factor is modified, while all other factors are not changed. Moreover, for analysis, no statistical programs are needed [21]. The method was applied by various groups for medium development [22–24].

Due to laborious experiments and lack of information about correlations between experiments or changed parameters, many other methods were established to optimize fermentations [21, 25]. Statistical methods are applied for medium development [16, 26, 27] and optimization of other crucial parameters, such as pH, temperature, and agitation rate [16, 26, 28]. By varying more than one component at a time and planning experiments strategically, process development is performed more efficiently than with classical methods, because the number of experiments can be reduced [21]. However, drawbacks of those methods are limitations due to a rigidly defined space for parameters to be investigated and local optima [29].

Müller et al. reported a new method, which was used to identify auxotrophies for a *Bacillus pumilus* strain in a chemically defined medium [19]. A systematic experimental procedure combined with online monitoring of the oxygen transfer rate (OTR) as indicator for metabolic behavior was applied. A nutrient-rich chemically defined medium, called modified Poolman medium, was used as starting point. The modified Poolman medium was based on a medium developed for *Lactobacillus* bacteria [30] and the V3 mineral medium, typically used for *Bacillus* species [31]. Auxotrophies were identified and medium components were reduced to essential ones by grouping the nutrients. Due to the combination with online techniques to monitor the respiration activity, no labor-intensive offline analysis of samples was necessary. However, the applicability of the method to identify growth limitations was not considered as nutrient concentrations were not investigated [19]. Lapierre et al. investigated the nutrient requirements for *Sporosarcina pasteurii* [32], using the systematic method developed by Müller et al. [19]. Essential nutrients and growth limitations were identified by systematically omitting or increasing nutrient groups [32]. As an alternative, online monitoring based on scattered light was performed instead of online monitoring of the OTR [32]. Biomass online monitoring via scattered light measurement can be influenced by cell morphology [33]. Therefore, changes in cell geometry due to sporulation of spore-forming microorganisms might affect the scattered light measurement.

The Respiration Activity MOnitoring System (RAMOS) enables the determination of the OTR, carbon dioxide transfer rate (CTR), and respiratory quotient (RQ) in eight parallel shake flasks over time in microbial cultivations [34, 35]. The respiration activity provides information about oxygen limitations [36], substrate limitations [37], product inhibitions [38], and diauxic growth [39]. As cultivations in microtiter plates (MTPs) enable process development in high-throughput, the µRAMOS was developed by Flitsch et al., which monitors the OTR in 48-well MTPs [40]. For example, the µRAMOS was used to perform high-throughput medium optimization for *B. pumilus* DSM 18097 [19] and mutagenicity tests [41].

*Paenibacillus polymyxa* is a facultative anaerobic and gram-positive bacterium and is only remotely related to *Bacillus subtilis* [42]. Under harsh conditions, *Paenibacillus* species can form free spores [42]. There are various potential applications for *Paenibacillus* species, such as in agriculture, medicine, bioremediation, and the production of 2,3-butanediol [43–46]. Additionally, *P. polymyxa* is an important strain for production of exopolysaccharides [47] that was even used to validate a device for non-invasive online monitoring of viscosity [48]. Most cultivations of *P. polymyxa* are performed in complex media [5, 46, 49, 50]. To our knowledge, only few mineral media, which were initially developed for other microbial strains, were used in the past for the cultivation of *P. polymyxa*. For example, Adlakha et al. cultivated *P. polymyxa* in a minimal medium adapted from Schepers et al. with 5 g/L glucose [51, 52]. Alvarez et al. used a chemically defined medium described in von der Weid et al. to investigate extracellular proteolytic enzyme production [53, 54]. However, none of those studies describes, which nutrients are essential for growth or enhance growth.

This study’s objective was to extend the systematic medium optimization method in combination with the µRAMOS technique [19] to identify growth limitations in chemically defined media, exemplarily for an industrial *P. polymyxa* strain. This strain was generated by random-mutagenesis of a wild-type isolate and designated as *P. polymyxa* JG1. In addition, the efficiency of the method of Müller et al. to reduce medium components [19] was confirmed. To best of our knowledge, this study is the first-time report of developing a defined medium for the industrially relevant *P. polymyxa* strain by using this medium optimization method*.* A screening procedure for *P. polymyxa* was established in MTPs and a reproduction of the cultivation in a laboratory fermenter was performed. The respiration activity of *P. polymyxa* showed good comparability in the µRAMOS and fermenter.

## Results

### Comparison of cultivation in complex and chemically defined media

To assess the performance of the applied chemically defined medium for growth of *P. polymyxa*, the strain was initially cultivated in a complex medium (here called *Paenibacillus polymyxa* (Pbp) complex medium) as reference. The composition is specified in Table 1. The chemically defined medium for *P. polymyxa* is called Moppa medium and is based on the modified Poolman medium after Müller et al. [19]. The composition of Moppa medium is specified in Table 2. However, while using the medium of Müller et al. [19] as a starting point, the carbon-, nitrogen-, phosphate source, and buffer, and their concentrations were adapted to the concentrations of main components present in the Pbp complex medium, listed in Table 1. Nutrient concentrations added by soy flour and yeast extract were not considered in this adaption. Additionally, the methionine concentration was increased to 200 mg/L (originally 125 mg/L) based on internal data (not published) that methionine is growth-relevant for the applied *Paenibacillus* strain. The biotin concentration was reduced from 3 mg/L to 0.06 mg/L, due to observations that another *P. polymyxa* strain showed sensibility to high biotin concentrations (internal data, not published). Furthermore, inositol was added to the Moppa medium, to test its relevance for growth of *P. polymyxa*.

**Table 1:** Composition of pre-culture and main-culture complex medium. The pre-culture complex medium contains 60 g/L maltose, and no buffer was used. The main-culture complex medium, called *Paenibacillus polymyxa* complex (Pbp) medium, contains 120 g/L maltose syrup instead of 60 g/L maltose, and additionally 0.1 M MES buffer. The letters in brackets indicate which components were prepared together in a stock solution. 3.21 g/L citric acid was added to the medium by the stock solution marked with (a) and 0.4 g/L was added by the stock solution marked with (c) to the final medium

**Ingredients**		**Concentration in the medium [g/L]**
**Main components**		
Maltose (Maltose syrup)		60 (120)
Citric acid * H_2_O	(a, c)	3.61
K_2_HPO_4_	(a)	1
(NH_4_)_2_SO_4_	(a)	1.07
MgSO_4_ * 7 H_2_O	(a)	1.62
Yeast extract	(a)	5
Soy flour	(a)	10
Antifoam	(a)	0.2
Ca(NO_3_)_2_ * 4 H_2_O		0.342
**Vitamins**		
Thiamine HCl	(b)	0.005
Nicotinic acid	(b)	0.005
Riboflavin	(b)	0.0002
Biotin	(b)	0.00005
Calcium pantothenate	(b)	0.001
Pyridoxine HCl	(b)	0.005
Vitamin B12	(b)	0.00005
Lipoic acid	(b)	0.00005
**Trace elements**		
MnSO_4_ * H_2_O	(c)	0.013
CuSO_4_ * 5 H_2_O	(c)	0.0046
Na_2_MoO_4_ * 2 H_2_O	(c)	0.0028
Fe_2_(SO_4_)_3_ * H_2_O	(c)	0.015

**Table 2:** Composition of chemically defined medium for *Paenibacillus polymyxa* (Moppa medium) for main-cultures and its variations. The letters in brackets indicate which components were prepared together in a stock solution. In experiments with varying concentrations of medium components, the preparation of stock solutions was adapted according to the varying components

**Ingredients**		**Concentration in Moppa medium [g/L]**	**Concentration in supplemented Moppa medium [g/L]**	**Concentration in reduced Moppa medium [g/L]**
**Main components**				
Maltose syrup		120	120	120
Citric acid * H_2_O	(a)	3.61	3.61	3.61
K_2_HPO_4_		1	3	3
(NH_4_)_2_SO_4_	(a)	1.07	3.21	3.21
MgSO_4_ * 7 H_2_O		1.62	1.62	1.62
MES buffer		19.5	39.0	39.0
Antifoam		0.2	0.2	0.2
Ca(NO_3_)_2_ * 4 H_2_O		0.342	0.342	0.342
**Vitamins**				
Ascorbic acid		0.5	0.5	
Biotin		0.00006	0.00006	0.00006
Nicotinic acid	(b)	0.0011	0.013	0.013
Calcium pantothenate	(b)	0.001	0.001	
p-aminobenzoic acid	(b)	0.01	0.01	
Pyridoxamine 2*HCl	(b)	0.006	0.006	
Pyridoxine HCl	(b)	0.002	0.002	
Vitamin B12	(b)	0.001	0.001	
Thiamine HCl	(b)	0.001	0.001	
Riboflavin		0.001	0.001	
Orotic acid		0.005	0.005	
Folic acid		0.001	0.001	
Inositol		0.0002	0.0002	
**Amino acids**				
L-Alanine		0.24	0.24	
L-Arginine		0.125	0.125	0.125
L-Aspartic acid		0.42	0.42	
L-Cysteine		0.13	0.13	
(S)-( +)-Glutamic acid		0.5	0.5	0.5
L-Glycine		0.175	0.175	
L-Histidine		0.15	0.15	0.15
L-Isoleucine		0.21	0.21	
L-Leucine		0.475	0.475	
L-Lysine		0.44	0.44	
L-Methionine		0.2	0.2	0.2
L-Phenylalanine		0.275	0.275	
L-Proline		0.675	0.675	0.675
L-Serine		0.34	0.34	
L-Threonine		0.225	0.225	
L-Tryptophane		0.05	0.05	
L-Tyrosine		0.25	0.25	
L-Valine		0.325	0.325	
**Nucleobases/ -sides**				
Adenine		0.01	0.01	
Guanine		0.01	0.01	
Inosine		0.005	0.005	
Xanthine		0.01	0.01	
Thymidine		0.005	0.005	
Uracil		0.01	0.01	
**Trace elements**				
ZnSO_4_ * 7 H_2_O	(c)	0.009	0.009	0.009
CoSO_4_ * 7 H_2_O	(c)	0.004	0.004	0.004
CuSO_4_ * 5 H_2_O	(c)	0.004	0.004	0.004
(NH_4_)_6_Mo_7_O_24_ * 4 H_2_O	(c)	0.003	0.003	0.003
CaCl_2_ * 2 H_2_O	(d)	0.066	0.066	0.066
MnCl_2_	(d)	0.016	0.016	0.016
FeCl_2_	(e)	0.005	0.005	0.005
FeCl_3_ * 6 H_2_O	(e)	0.005	0.005	0.005

**Fig. 1:**
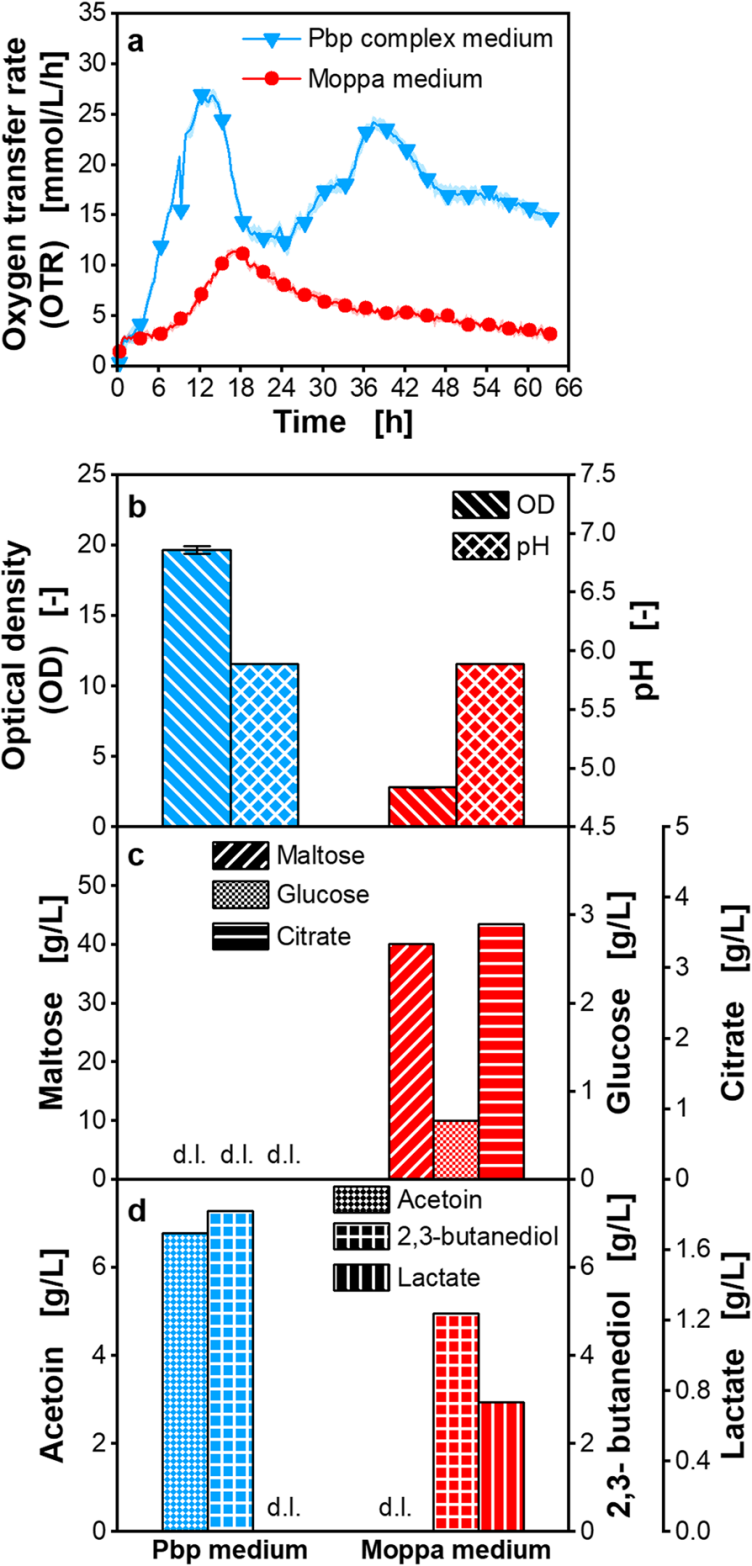
Cultivation of *Paenibacillus polymyxa* in complex medium and chemically defined Moppa medium in microtiter plate. Pbp complex medium (specified in Table 1) or chemically defined Moppa medium (specified in Table 2)*.* Initial concentrations were: 56.2–56.8 g/L maltose, 3.5–3.7 g/L glucose, 3.1–3.4 g/L citrate. **a** Oxygen transfer rate (OTR), **b** Final optical density (OD) and pH, **c** Final maltose, glucose and citrate concentration, **d **Final acetoin, 2,3-butanediol and lactate concentration. **a** For clarity, only every 10th measuring point over time is marked as a symbol. Mean values for OTR of at least four replicates with standard deviations as shadows are shown. Standard deviations are not well recognizable, because they are small. **b**-**d** For offline analysis, samples (wells) of the replicates of the OTR measurement were pooled at the end of the experiments. OD measurement of pooled samples was performed in triplicate and mean values with standard deviations depicted as error bars are shown. pH and concentrations of sugars and metabolites were determined in a single measurement of pooled samples. **c**, **d** d.l. means that concentrations of components were lower than the detection limit. Parameters in b-d were determined after 63.3 h. **b** OD of complex medium prior to inoculation (3.6) is not considered in the final measured optical density. Initial osmolality in Pbp medium is 0.63 osmol/kg and osmolality after 63.3 h is 0.57 osmol/kg. Initial osmolality in Moppa medium is 0.69 osmol/kg and osmolality after 63.3 h is 0.73 osmol/kg. Cultivation conditions: temperature 33 °C, 48-round well plate, filling volume 0.8 mL, shaking frequency 1000 rpm, shaking diameter 3 mm, 0.1 M MES, initial pH 6.5

To compare the growth performance in the Pbp complex medium and the Moppa medium, the respiration activity, optical density (OD), pH, carbon source consumption, and overflow metabolite production of *P. polymyxa* were evaluated and are shown in Fig. 1. The cultivations were performed at least in quadruplicates in the µRAMOS, and mean values with standard deviation as shadows are shown for the OTR (Fig. 1a). The low standard deviations (on average < 8.5%) showed the good reproducibility of the measuring device and the metabolic activity of *P. polymyxa* in a single experiment. The offline values were determined after combining the content of at least four wells of the MTP.

During cultivations of *P. polymyxa* in complex medium, the course of the OTR showed three distinct peaks at 21 mmol/L/h after 9.0 h, at 27 mmol/L/h after 12.3 h, and at 24 mmol/L/h after 37.3 h (Fig. 1a). An OD of 19.7 after 63.3 h of cultivation was achieved (Fig. 1b). However, it must be considered that the complex medium itself had an initial OD of 3.6 prior to inoculation, as soy flour is not completely soluble. Citrate, as well as the carbon sources maltose and free glucose of the maltose syrup, were completely consumed after 63.3 h (Fig. 1c). The pH of the medium decreased to 5.9 after 63.3 h of cultivation, which was lower than the initial pH of 6.5 (Fig. 1b). Lactate was not detected after 63.3 h. An acetoin and 2,3-butanediol concentration of 6.8 g/L and 7.3 g/L, respectively, were measured (Fig. 1d).

The fluctuating course of the OTR profile (Fig. 1a) represents the metabolic activity of *P. polymyxa* during growth on various carbon sources of the Pbp complex medium, such as sugars and proteins from soy flour and yeast extract, but also from maltose syrup, and citrate. Additionally, in the later phase of the cultivation, consumption of produced overflow metabolites is possible. However, the undefined composition of the medium complicates the interpretation of the OTR peaks, indicating the necessity of applying a chemically defined medium.

In Fig. 1, the growth of *P. polymyxa* in Pbp complex medium was compared to the chemically defined Moppa medium. The OTR peak reached in the chemically defined medium was 11 mmol/L/h after 18.0 h (Fig. 1a); no additional peaks were observed. This peak value was 2.5-fold lower than in the complex medium. A statistically significant difference of the peak values in chemically defined medium after 18.0 h and in complex medium after 12.3 h was shown (Additional file 1: Table S1). The growth rate, determined based on the slope of the logarithm of the OTR in Moppa medium, was 0.12 1/h compared to 0.29 1/h in Pbp complex medium (Additional file 1: Fig. S1).

The low respiration activity in Moppa medium is confirmed by the offline data. The OD reached only 2.8 in Moppa medium compared to 19.7 in Pbp complex medium after 63.3 h (Fig. 1b). In contrast to the cultivation in complex medium, residual maltose, glucose, and citrate remained in Moppa medium after 63.3 h (Fig. 1c). A lower amount of carbon source is consumed, due to obvious nutritional limitations in this medium. This resulted in lower concentrations of produced acetoin and 2,3-butanediol (Fig. 1d). Only the final pH values were comparable (Fig. 1b). In conclusion, the results in Fig. 1 clearly indicate a limited growth of *P. polymyxa* in the chemically defined Moppa medium compared to the Pbp complex medium. Therefore, one or more nutrient limitations and growth inhibitions in the Moppa medium can be assumed. The nutritional limitations and growth inhibitions in the medium were systematically identified in the next step.

### Identificatio﻿n of growth limitations and inhibitions in the chemically defined Moppa medium

A schematic overview of the experiments to identify growth limitations and inhibitions can be found in Additional file 1: Fig. S2. First, the nutrient groups were defined: amino acids, nucleobases/-sides, vitamins, and trace elements (Additional file 1: Fig. S2). Nutrients added in similar concentrations to both, the Moppa medium and Pbp complex medium, were not considered potentially limiting. These nutrients were: maltose syrup, citrate, K_2_HPO_4_, (NH_4_)_2_SO_4_, MgSO_4_, and Ca(NO_3_)_2_. The amino acids used in the Moppa medium were divided into six groups, as specified in Additional file 1: Table S2. The division was adapted from the work of Müller et al. [19], based on the amino acid metabolism of *Escherichia coli* and *B. subtilis* [55], according to the KEGG database. The grouping was based on intermediates or their precursors from glycolysis, citric acid cycle, or pentose phosphate pathway. Methionine was assigned to a separate group, based on internal data (not published) that it is growth-relevant for the applied *Paenibacillus* strain in this study. The vitamin grouping was also performed after Müller et al. [19], with some modifications. It is specified in Additional file 1: Table S3. Biotin was assigned to a separate group, due to the observations that another *P. polymyxa* strain showed sensibility to high biotin concentrations (internal data, not published). Inositol was added to vitamin group 3.

To identify the limiting nutrient group(s), the concentration of each nutrient group was increased (Additional file 1: Fig. S3). Biotin was not raised, as the suggested sensitivity could overlay growth-enhancing effects of the other vitamins (Additional file 1: Fig. S3a). Due to practical issues and the high number of amino acids, they were increased in single groups (Additional file 1: Fig. S3b). Only the increase of the vitamins resulted in a higher OTR peak (Additional file 1: Fig. S3a). Therefore, the next step was to identify the limiting vitamin(s).

In parallel cultivations, the concentrations of each single vitamin group were increased, to identify the possible limitation(s) (Additional file 1: Fig. S4). In these experiments, the concentration of biotin (vitamin group 4) was also increased. The cultivation with the three-fold higher concentration of vitamin group 1 resulted in a higher OTR peak of 19 mmol/L/h compared to 11 mmol/L/h in the cultivation with the one-fold concentration of all vitamins (Additional file 1: Fig. S4a). The cultivations with increased concentrations of all other vitamin groups resulted in OTR courses similar to the cultivation with the one-fold concentration of all vitamins. There was no sensitivity observed at increased biotin concentrations for the strain of this study, as no growth inhibition was detected. The results of online monitoring of the OTR are in agreement with offline data (Additional file 1: Fig. S4b, c and d). In the cultivation with the three-fold concentration of vitamin group 1, the OD and maltose consumption after 63.3 h was 2.1-fold and 1.1-fold, respectively, higher than in the cultivation with the one-fold concentration of all vitamins (Additional file 1: Fig. S4b and c). The 2,3-butanediol production was 1.2-fold lower and the lactate concentration was below the detection limit (Additional file 1: Fig. S4d). It can be concluded that at least one vitamin of group 1 is limiting in the Moppa medium.

Since the growth limitation was narrowed down to vitamin group 1, the next step was to increase the concentration of every single vitamin of this group in parallel cultivations (Additional file 1: Fig. S5). Only the increase of the nicotinic acid concentration resulted in a higher respiration activity (Additional file 1: Fig. S5a) and OD value after 66.3 h of cultivation (Additional file 1: Fig. S5b).

**Fig. 2:**
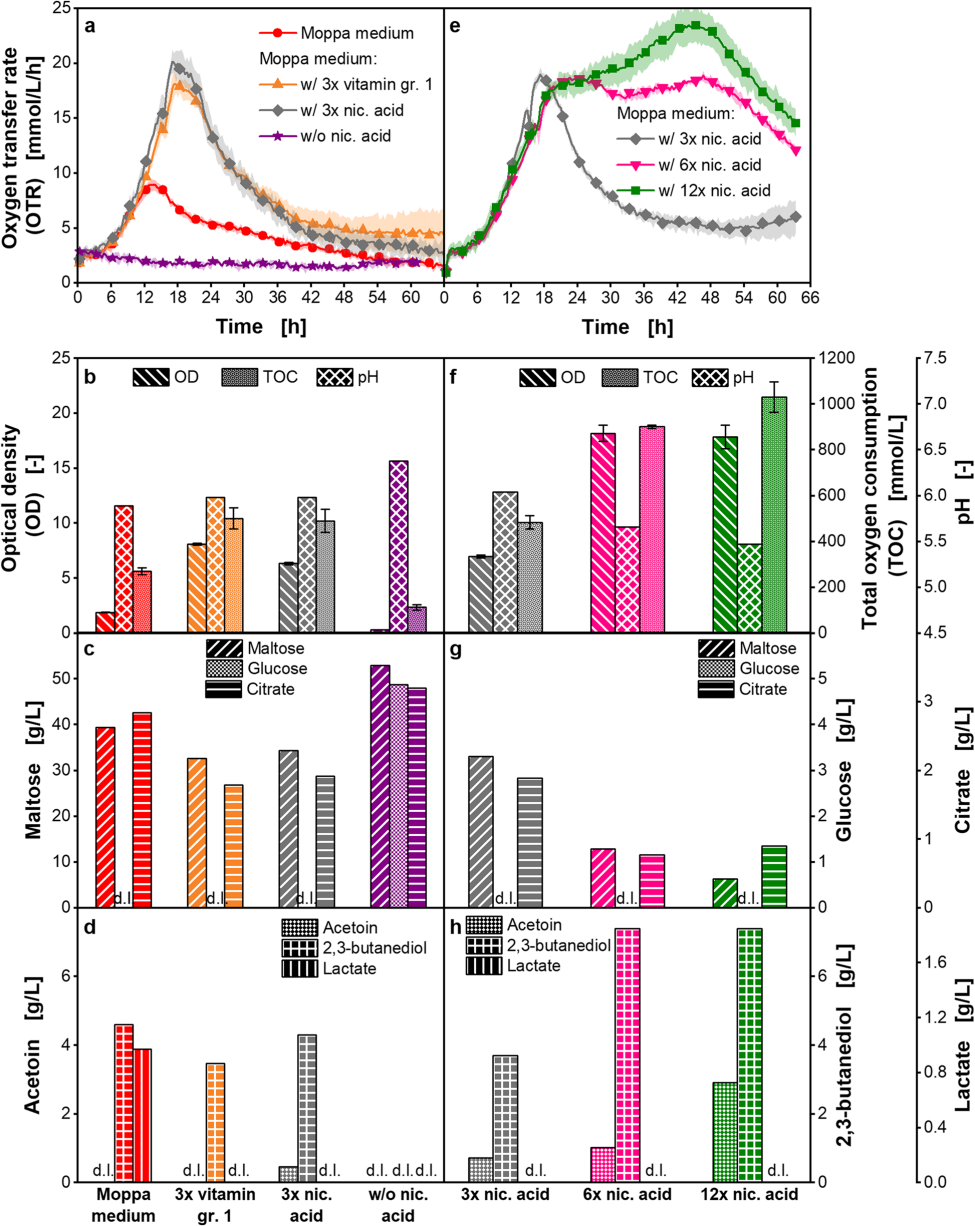
Cultivation of *Paenibacillus polymyxa* with increased concentrations of nicotinic acid in microtiter plate. Moppa medium (specified in Table 2) without or with increased concentrations of all vitamins of group 1 or of nicotinic acid. Vitamin group (gr.) 1 is specified in Additional file 1: Table S3. nic.: nicotinic*.* Initial concentrations were: 54.2–56.3 g/L (**a**-**d**) and 51.8–53.1 g/L (**e**–**h**) maltose, 4.2–4.5 g/L (**a**-**d**) and 4.1–4.3 g/L (**e**–**h**) glucose, 3.3–3.4 g/L (**a**-**d**) and 3.2–3.6 g/L (**e**–**h**) citrate. **a**, **e**: Oxygen transfer rate (OTR), **b**, **f**: Final optical density (OD), total oxygen consumption (TOC) and pH, **c**, **g**: Final maltose, glucose and citrate concentration, **d**, **h**: Final acetoin, 2,3-butanediol and lactate concentration. **a**, **b**: For clarity, only every 10th measuring point over time is marked as a symbol. Mean values for OTR of at least three replicates with standard deviations as shadows are shown. **b**, **f**: TOC was determined based on OTR data of the replicates. Mean values for TOC of the replicates with standard deviations depicted as error bars are shown. **b**-**h**: For offline analysis, samples (wells) of the replicates of the OTR measurement were pooled at the end of the experiments. OD measurement of pooled samples was performed in triplicate and mean values with standard deviations depicted as error bars are shown. OD of pooled sample of the cultivation w/o nicotinic acid was measured in duplicate and the mean value without standard deviation is shown. pH and concentrations of sugars and metabolites were determined in a single measurement of pooled samples. **c** – **h**: d.l. means that concentrations of components were lower than the detection limit. Parameters in c – h were determined after 61 – 66 h. Cultivation conditions: temperature 33 °C, 48-round well plate, filling volume 0.8 mL, shaking frequency 1000 rpm, shaking diameter 3 mm, 0.1 M MES, initial pH 6.5

The influence of different nicotinic acid concentrations on growth is shown in more detail in Fig. 2. The cultivations with the one-fold concentration of all medium components, in the legend referred to as Moppa medium, the three-fold concentration of vitamin group 1, and the three-fold concentration of nicotinic acid are shown in Fig. 2 a, b, c and d. Similar courses of the OTR over time and total oxygen consumption (TOC), OD values, maltose, and citrate consumption after 66.3 h were observed for the cultivations with the three-fold nicotinic acid concentration and the three-fold concentration of vitamin group 1 (Fig. 2a, b and c). The OTR peak of the cultivation with the three-fold nicotinic acid concentration was slightly higher than the OTR peak of the cultivation with the three-fold concentration of vitamin group 1. A statistically significant difference of the OTR peaks was shown (Additional file 1: Table S4). The OTR peak was about two-fold higher for both cultivations than for the cultivation with the one-fold concentration of all components. This difference of the OTR peaks was statistically significant (Additional file 1: Table S4).

In the cultivations with the one-fold concentration of all medium components, the three-fold concentration of vitamin group 1, and the three-fold concentration of nicotinic acid, a pH drop of around 0.5 pH units was observed (Fig. 2b). In addition, acetoin was produced in final concentrations lower than 1.0 g/L (Fig. 2d). 2,3-butanediol concentrations were at similar levels of around 4.0 g/L in all cultivations, despite the increased nicotinic acid concentration (Fig. 2d). Lactate was only detected in the cultivation with the one-fold concentration of all components (Fig. 2d). Possibly, the *Paenibacillus* strain consumed the produced lactate in the other cultivations.

Nicotinic acid was identified as the growth limiting vitamin of group 1. When nicotinic acid was left out of the medium, no growth of *P. polymyxa* was possible (Fig. 2a, b, c and d). Maltose, glucose, and citrate concentrations were on comparable levels at the beginning and at the end of the cultivation (Fig. 2c). As no growth was observed in the cultivation without nicotinic acid, this vitamin is absolutely essential for the applied *P. polymyxa* strain.

In Fig. 2a, the OTR peak at 20 mmol/L/h of the cultivation in Moppa medium with the three-fold nicotinic acid concentration is still significantly lower than the OTR peak at 27 mmol/L/h in the cultivation using complex medium (Fig. 1) (t-test, p-value < 0.001). Therefore, the nicotinic acid concentration was increased six-fold and 12-fold (Fig. 2e, f, g and h).

The cultivations with the six-fold and 12-fold nicotinic acid concentration showed an OTR peak after 22.3 h. Those peaks were on a comparable level as the peak of the cultivation with the three-fold nicotinic acid concentration after 17.3 h. There was no statistically significant difference of the OTR peaks (ANOVA, p-value = 0.484). Hence, the six-fold concentration of nicotinic acid did not increase the OTR peak, compared to the cultivation with the three-fold concentration (Fig. 2e). Instead, after the first peak at 22.3 h, a plateau in the OTR course was observed. For the cultivation with the 12-fold nicotinic acid concentration, a low increase of the OTR was visible after the OTR peak at 22.3 h. This indicates a limitation by a secondary substrate or a growth inhibition in the medium.

The TOC increased from 484 mmol/L in the cultivation with the three-fold nicotinic acid concentration to 901 mmol/L for the six-fold and 1031 mmol/L for the 12-fold concentration of nicotinic acid (Fig. 2f). Additionally, the OD after 63.3 h was 2.6-fold higher for both cultivations with increased nicotinic acid concentration than for the cultivation with the three-fold nicotinic acid concentration (Fig. 2f).

*P. polymyxa* was metabolically active for a longer cultivation time when the nicotinic acid concentration was increased from three-fold to six- and 12-fold. Additionally, the increase of the nicotinic acid concentration from three-fold to six-fold resulted in a higher biomass formation, represented in the OD (Fig. 2f), and in the production of higher concentrations of acetoin and 2,3-butanediol (Fig. 2h). The increase of the nicotinic acid concentration from six-fold to 12-fold did not result in a higher OD and 2,3-butanediol concentration (Fig. 2f and h). Only a higher acetoin production was observed (Fig. 2h). Lactate as a potential metabolic side product was not detected via high-performance liquid chromatography (HPLC) in any sample taken after 63.3 h.

After 63.3 h of cultivation, the concentration of citrate was 2.4-fold and 2.1-fold lower for the cultivations with the six- and 12-fold nicotinic acid concentration, respectively, than in the cultivation with the three-fold nicotinic acid concentration (Fig. 2g). This higher consumption rate of citrate (Fig. 2g) might be explained by an increased activity of the citric acid cycle due to elevated glycolysis.

Increasing the nicotinic acid concentration from one- to three-fold resulted in a similar pH value after 66.3 h (Fig. 2b). Hence, the buffer capacity for the applied cultivation conditions was sufficient. A further increase of the nicotinic acid concentration resulted in a more substantial decrease of pH values (Fig. 2f). In the cultivations with the six- and 12-fold nicotinic acid concentration, a pH of 5.7 and 5.5 was measured after 63.3 h, respectively (Fig. 2f). Therefore, in contrast to the cultivations with one- and three-fold nicotinic acid concentrations, an insufficient buffer capacity is assumed for the six- and 12-fold nicotinic acid concentrations. This might result in a subsequent growth inhibition in the cultivation.

**Fig. 3:**
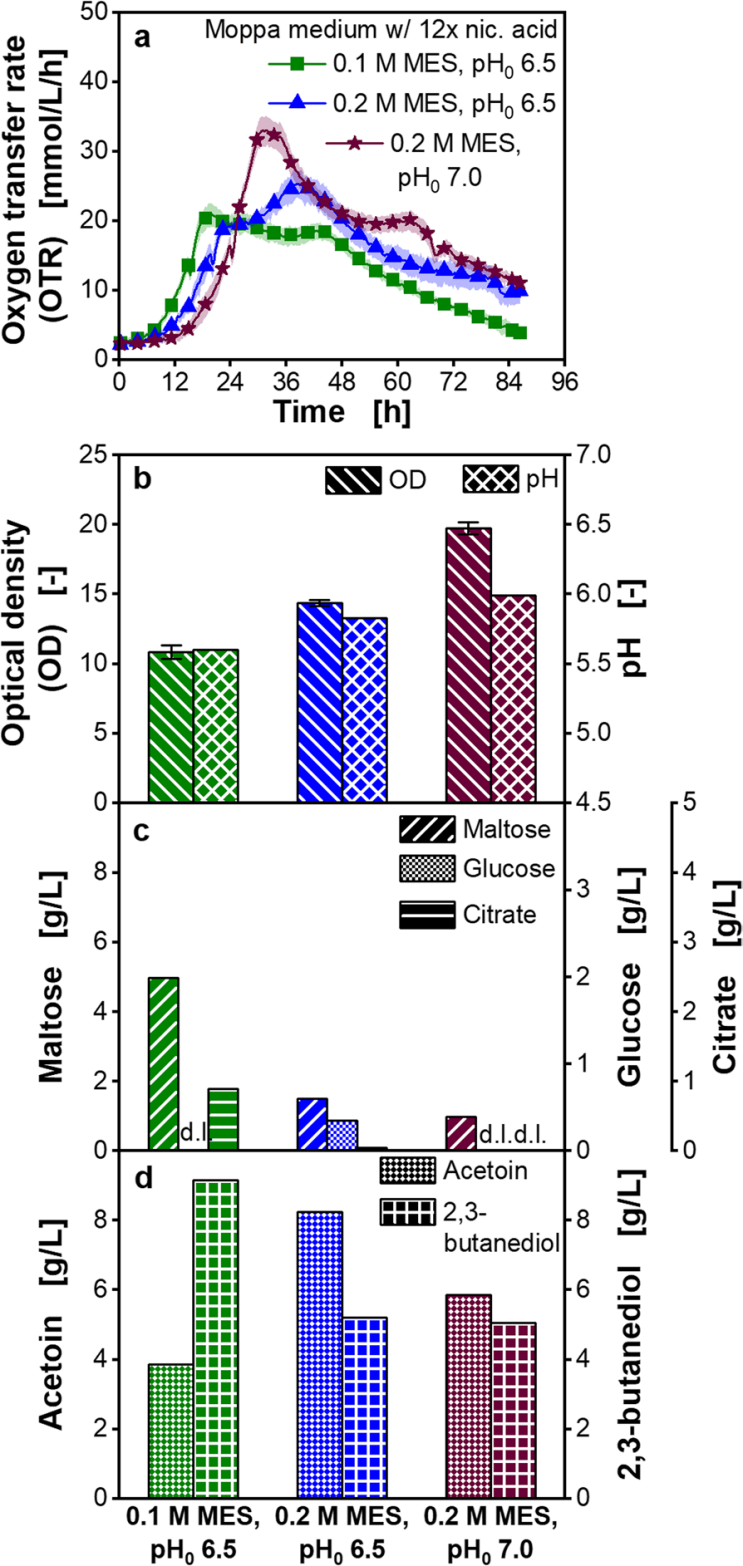
Cultivation of *Paenibacillus polymyxa* with increased pH — buffer capacity in microtiter plate. Moppa medium (specified in Table 2) with 12 × nicotinic acid or Moppa medium with 12 × nicotinic acid and with increased buffer concentration or with increased buffer concentration and initial pH. nic.: nicotinic, pH_0_: initial pH*.* Initial concentrations were: 62.1–63.4 g/L maltose, 1.9–2.1 g/L glucose, 3.7–3.8 g/L citrate. **a** Oxygen transfer rate (OTR), **b** Final optical density (OD) and pH, **c** Final maltose, glucose and citrate concentration, d: Final acetoin and 2,3-butanediol concentration. **a** For clarity, only every 12th measuring point over time is marked as a symbol. Mean values for OTR of at least four replicates with standard deviations as shadows are shown. **b**-**d** For offline analysis, samples (wells) of the replicates of the OTR measurement were pooled at the end of the experiments. OD measurement of pooled samples was performed in triplicate and mean values with standard deviations depicted as error bars are shown. pH and concentrations of sugars and metabolites were determined in a single measurement of pooled samples. **c** d.l. means that concentrations of components were lower than the detection limit. Final lactate concentrations were lower than the detection limit. Parameters in c-d were determined after 86.3 h. Osmolalities are shown in Additional file 1: Figure S6. Cultivation conditions: temperature 33 °C, 48-round well plate, filling volume 0.8 mL, shaking frequency 1000 rpm, shaking diameter 3 mm

To verify this hypothesis, the buffer capacity in the MTP cultivations was increased (Fig. 3). The Moppa medium with the 12-fold nicotinic acid concentration was used. The pK_a_ value of 2-(*N*-morpholino)ethanesulfonic acid (MES) buffer is 6.0 at a cultivation temperature of 33 °C [56]. Therefore, the initial pH was increased, to better exploit the buffer capacity. Additionally, the buffer concentration was increased from 0.1 to 0.2 M MES.

The OTR peak was increased from 21 mmol/L/h in the cultivation with 0.1 M MES and an initial pH of 6.5 to a value of 33 mmol/L/h in the cultivation with 0.2 M MES and an initial pH of 7.0 (Fig. 3a). A statistically significant difference of the OTR peaks was observed (Additional file 1: Table S5). Additionally, a higher OD was achieved (Fig. 3b), which is also represented in higher consumption of maltose and citrate (Fig. 3c). The acetoin concentration increased to 8.3 g/L in the cultivation with 0.2 M MES with an initial pH of 6.5 compared to 3.9 g/L in the cultivation with the lowest buffer capacity. A further increase of the buffer capacity (0.2 M MES, initial pH 7.0) resulted in a decreased acetoin concentration (Fig. 3d). 2,3-butanediol concentrations decreased from 9.2 g/L in the cultivation with 0.1 M MES and an initial pH of 6.5 to a concentration of around 5.2 g/L in both cultivations with higher buffer capacity (Fig. 3d).

The lag-phase using 0.2 M MES buffer and an initial pH of 7.0 increased by 4.7 h (Additional file 1: Fig. S6) compared to the cultivation with 0.1 M MES and an initial pH of 6.5. An increase of the initial osmolality from 0.68 osmol/kg, which is similar to the osmolality in complex medium, to 0.94 osmol/kg using 0.2 M MES with an initial pH of 7.0 (Additional file 1: Fig. S6) was observed.

The salt solutions (K_2_HPO_4_, (NH_4_)_2_SO_4_, MgSO_4_, Ca(NO_3_)_2_) and their concentrations in the chemically defined Moppa medium (Table 2), were similar to those added in the Pbp complex medium (Table 1). Therefore, only amino acids, nucleobases/-sides, vitamins, and trace elements were considered in the previous experiments. However, salts are added to the Pbp medium by the salt solutions itself, soy flour and yeast extract. To exclude that any salts, like the nitrogen and phosphate source are limiting in the Moppa medium, the composition of the elements of salts was calculated (Additional file 1: Fig. S7a). The nitrogen (N), magnesium (Mg), phosphorous (P), sulfur (S), and potassium (K) concentrations in the Moppa medium with the 12-fold nicotinic acid concentration were calculated per amount of carbon source (C) in mol/mol (Additional file 1: Fig. S7a). Those elemental compositions were compared to other proven media known from literature for *Paenibacillus* and *Bacillus* species [19, 31, 51], *E. coli* [57], and lactic acid bacteria [30]. Compared to the other media, all elements (N, P, S, K), except Mg, were available in significantly lower amounts per amount of carbon in the Moppa medium (Additional file 1: Fig. S7a). Additionally, the elemental composition of trace elements was considered (Additional file 1: Fig. S7b). The comparison of the elemental composition of trace elements in Moppa medium compared to the other literature known media showed that those elements are available in sufficient amounts in Moppa medium (Additional file 1: Fig. S7b). Due to the low amount per carbon of N, P, S, and K in Moppa medium compared to the other media, cultivations with increased concentrations of (NH_4_)_2_SO_4_ and K_2_HPO_4_ were performed (Additional file 1: Fig. S8). For this purpose, Moppa medium with the 12-fold nicotinic acid concentration, 0.2 M MES buffer, and an initial pH of 7.0 was used. Respiration activities, ODs, final pH values, and 2,3-butanediol concentrations were comparable for all cultivations (Additional file 1: Fig. S8a, b, and d). Maltose and citrate were consumed entirely (Additional file 1: Fig. S8c). Only slight deviations in glucose and acetoin concentrations were observed (Additional file 1: Fig. S8c and d).

To avoid any limitations in fermenter experiments and to avoid a nitrogen limitation by reducing the amino acids to the growth-relevant ones, the Moppa medium with additional (NH_4_)_2_SO_4_ and K_2_HPO_4_ was used for further experiments. From here on, the Moppa medium with the 12-fold nicotinic acid concentration, 0.2 M MES, an initial pH of 7.0, and the three-fold (NH_4_)_2_SO_4_ and K_2_HPO_4_ concentration is called supplemented Moppa medium.

### Reduction of medium components to growth relevant ones

Comparing the OTR peak of 35 mmol/L/h in the cultivation with supplemented Moppa medium (Additional file 1: Fig. S8a) with the OTR peak of 27 mmol/L/h in the cultivation with Pbp complex medium (Fig. 1a), the OTR peak in the cultivation with supplemented Moppa medium was higher. However, the supplemented Moppa medium contains 53 components compared to 22 components in the complex medium. A high number of medium components leads to laborious medium preparation and high medium costs. Therefore, the medium components were reduced to the essential and growth-relevant ones. A schematic overview of the experiments performed in order to reduce the medium components is shown in Additional file 1: Fig. S9. A systematic approach by grouping the nutrients was chosen.

Preliminary experiments were performed to reduce the vitamins in Moppa medium with the three-fold nicotinic acid concentration, 0.1 M MES, an initial pH of 6.5, and the one-fold concentration of (NH_4_)_2_SO_4_ and K_2_HPO_4_. An OTR peak of 15 mmol/L/h was achieved after 55.0 h in the cultivation without biotin compared to 19 mmol/L/h after 17.3 h in the cultivation with biotin (Additional file 1: Fig. S10). Leaving the other vitamins, listed in Additional file 1: Table S3, out from the medium, did not influence the respiration activity (data not shown). The results show that the *P. polymyxa* strain can grow without biotin in the medium, but the OTR peak is reached with a substantial time delay. Therefore, biotin enhances growth of *P. polymyxa*, but is not absolutely essential for the strain.

**Fig. 4:**
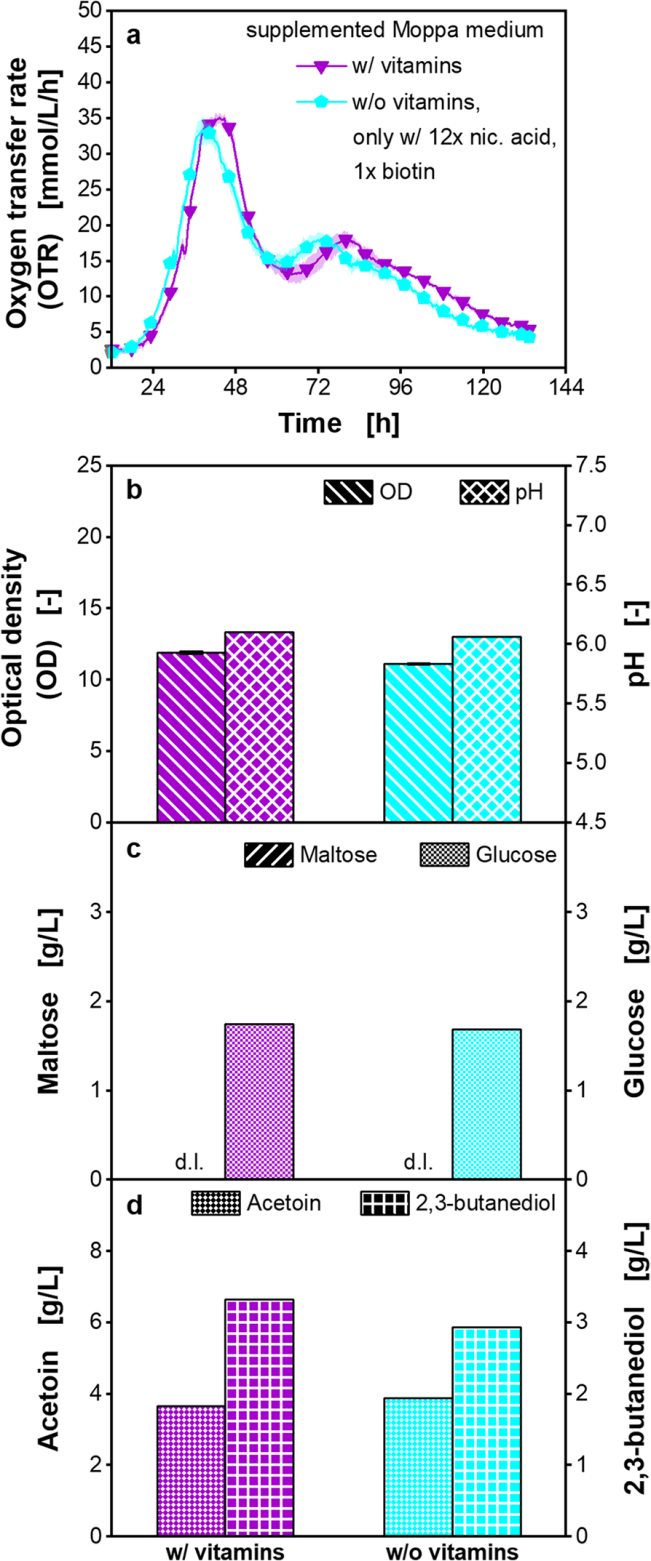
Cultivation of *Paenibacillus polymyxa* only with growth relevant vitamins in microtiter plate. Supplemented Moppa medium (specified in Table 2) with or without vitamins (only with nicotinic acid and biotin). nic.: nicotinic. Initial concentrations were: 56.6–57.0 g/L maltose, 3.2–3.3 g/L glucose, 3.0 g/L citrate. **a** Oxygen transfer rate (OTR), **b** Final optical density (OD) and pH, **c** Final maltose and glucose concentration, **d** Final acetoin and 2,3-butanediol concentration. **a** For clarity, only every 18th measuring point over time is marked as a symbol. Mean values for OTR of four replicates with standard deviations as shadows are shown. Standard deviations are not well recognizable, because they are small. **b**-**d** For offline analysis, samples (wells) of the replicates of the OTR measurement were pooled at the end of the experiments. OD measurement of pooled samples was performed in triplicate and mean values with standard deviations depicted as error bars are shown. pH and concentrations of sugars and metabolites were determined in a single measurement of pooled samples. **c** d.l. means that concentrations of components were lower than the detection limit. Final citrate and lactate concentrations were lower than the detection limit. Parameters in b-d were determined after 133.3 h. Cultivation conditions: temperature 33 °C, 48-round well plate, filling volume 0.8 mL, shaking frequency 1000 rpm, shaking diameter 3 mm

Based on these results, a cultivation in supplemented Moppa medium was performed, adding only nicotinic acid and biotin as vitamins. All other vitamins listed in Additional file 1: Table S3 were left out of the medium (Fig. 4). All measured parameters, the OTR course over time, the OD, the pH, and the sugar and metabolite concentrations after 133.3 h were similar to the cultivation with the addition of all vitamins. There was no statistically significant difference of the OTR peaks (Additional file 1: Table S6). The results show that only nicotinic acid and biotin are growth-relevant vitamins.

**Fig. 5:**
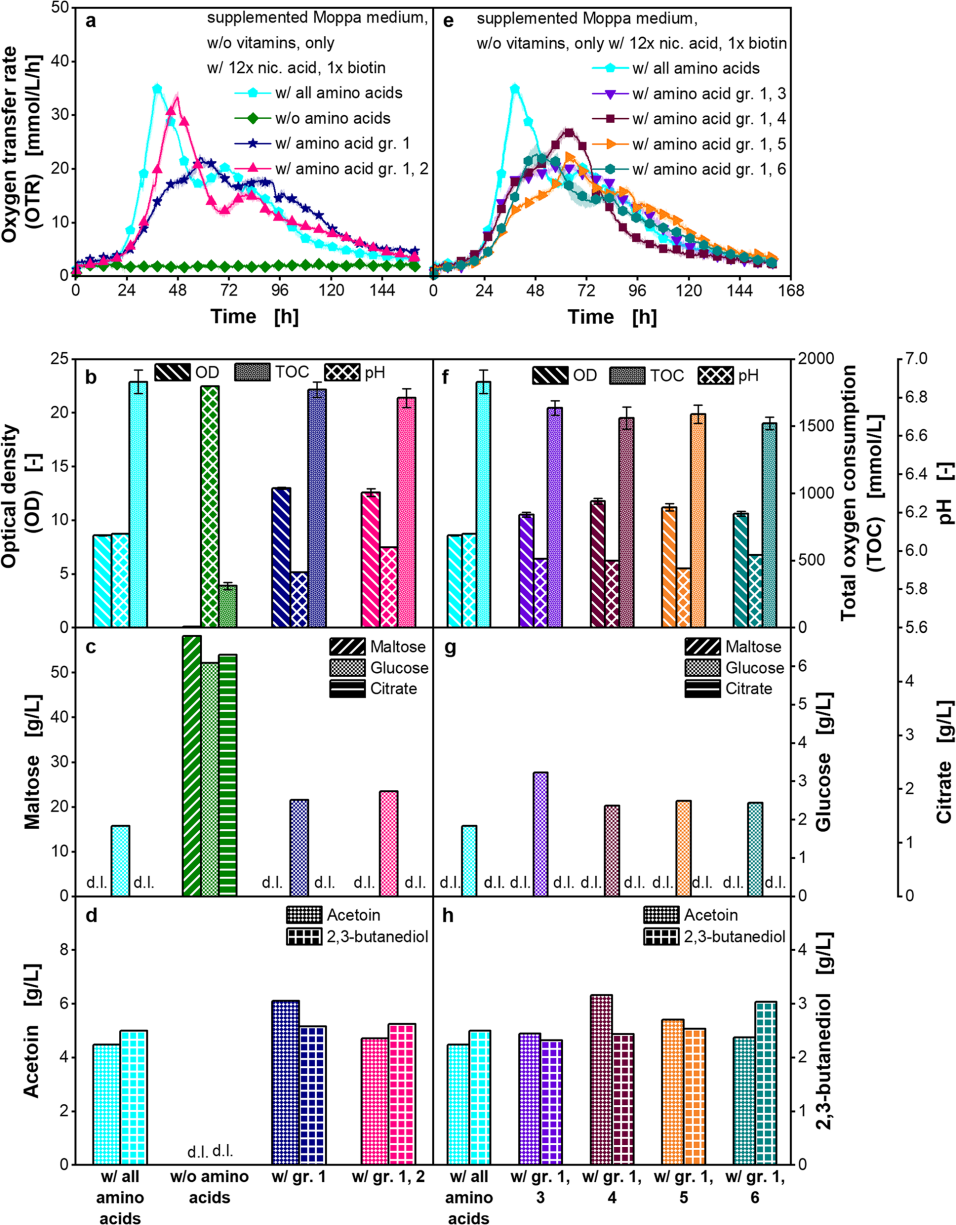
Cultivation of *Paenibacillus polymyxa* with varying amino acid composition in microtiter plate. Supplemented Moppa medium (specified in Table 2) without vitamins (only with nicotinic acid and biotin) and with or without amino acid groups. nic.: nicotinic. Amino acid groups (gr.) are specified in Additional file 1: Table S2. Initial concentrations were: 54.1–56.3 g/L (**a**-**d**) and 54.1–56.1 g/L (**e**–**h**) maltose, 2.9–3.2 g/L (**a**-**d**) and 2.9–3.1 g/L (**e**–**h**) glucose, 3.0–3.1 g/L (**a**-**d**) and 3.0–3.1 g/L (**e**–**h**) citrate. **a**, **e**: Oxygen transfer rate (OTR), **b**, **f**: Final optical density (OD), total oxygen consumption (TOC) and pH, **c**, **g**: Final maltose, glucose and citrate concentration, **d**, **h**: Final acetoin and 2,3-butanediol concentration. **a**, **b**: For clarity, only every 20th measuring point over time is marked as a symbol. Mean values for OTR of at least three replicates with standard deviations as shadows are shown. **b**, **f**: TOC was determined based on OTR data of the replicates. Mean values for TOC of the replicates with standard deviations depicted as error bars are shown. **b**-**h**: For offline analysis, samples (wells) of the replicates of the OTR measurement were pooled at the end of the experiments. OD measurement of pooled samples was performed in triplicate and mean values with standard deviations depicted as error bars are shown. OD of pooled sample of the cultivation w/o amino acids was measured in duplicate and the mean value without standard deviation is shown. pH and concentrations of sugars and metabolites were determined in a single measurement of pooled samples. **c**, **d**, **g**: d.l. means that concentrations of components were lower than the detection limit. Final lactate concentrations were lower than the detection limit. Parameters in b-d and f–h were determined after 159.3 **h**. Cultivation conditions: temperature 33 °C, 48-round well plate, filling volume 0.8 mL, shaking frequency 1000 rpm, shaking diameter 3 mm

In the next step, the amino acids were investigated. Therefore, only nicotinic acid and biotin were added as vitamins to the supplemented Moppa medium. The amino acids were divided into six groups, specified in Additional file 1: Table S2. All amino acids were left out from the medium, while every single group was added (Fig. 5). The *P. polymyxa* strain could not grow in a medium without any supplemented amino acid. By adding amino acid group 1, which contained only methionine, growth was possible, and an OTR peak at 22 mmol/L/h was observed (Fig. 5a). Hence, methionine is an essential amino acid for the *P. polymyxa* strain.

In the cultivation supplemented with amino acid group 1, the OTR peak (Fig. 5a) and the growth rate (Additional file 1: Fig. S11) were 1.6-fold and 1.8-fold lower, respectively, than in the cultivation with supplementation of all amino acids. The difference of the OTR peaks was statistically significant (Additional file 1: Table S7). Nevertheless, the TOC was at comparable levels. In the cultivation supplemented with amino acid group 1, 1773 mmol/L oxygen was consumed, and in the cultivation supplemented with all amino acids, 1832 mmol/L was consumed (Fig. 5b). In contrast, the OD in the cultivation with amino acid group 1 was 1.5-fold higher than that in the cultivation with all amino acids (Fig. 5b).

Maltose and citrate were entirely consumed in both cultivations, and around 2–3 g/L glucose was measured at the end of the cultivations (Fig. 5c). 2,3-butanediol concentrations were on comparable levels after 159.3 h, and the acetoin concentration was 1.4-fold higher in the cultivation with only amino acid group 1 than in the cultivation with all amino acids (Fig. 5d). The pH after 159.3 h was lower in the cultivation with only amino acid group 1 than in the cultivation containing all amino acid groups (Fig. 5b). Possibly a higher amount of produced acetate, which was not measured in this study, led to the lower final pH in the cultivation containing only amino acid group 1.

The OTR course of the cultivation containing only amino acid group 1 is not entirely comparable to the OTR course of the cultivation containing all amino acids (Fig. 5a). Hence, it was supposed that at least one amino acid was missing. To identify the limiting amino acid group, an individual supplementation of each group to the medium already containing amino acid group 1 was performed.

The addition of amino acid groups 1 and 2 resulted in a comparable OTR peak as in the cultivation containing all amino acid groups (Fig. 5a). There was no statistically significant difference of the OTR peaks (Additional file 1: Table S7). However, the peak was measured 9.3 h later than in the cultivation with all amino acids. This delay was reflected in a slightly lower growth rate, which was 0.14 1/h in the cultivation with all amino acids and 0.10 1/h in the cultivation with only amino acid groups 1 and 2 (Additional file 1: Fig. S11). Adding only amino acid groups 1 and 2 to the cultivation medium, the observed OTR peak increased 1.5-fold compared to the cultivation with only amino acid group 1 (Fig. 5a). A statistically significant difference of the OTR peaks was observed (Additional file 1: Table S7). Offline data were similar (Fig. 5 b, c and d).

The addition of amino acid group 3, 5, or 6 together with amino acid group 1 did not significantly increase the respiration activity compared to the cultivation with only amino acid group 1 (Fig. 5e, Additional file 1: Table S7). The addition of amino acid group 4 together with amino acid group 1 resulted in a slightly higher OTR peak, compared to the addition of only amino acid group 1. A statistically significant difference of these two OTR peaks was shown (Additional file 1: Table S7). However, the TOC was on comparable levels. The results were also reflected in the offline data (Fig. 5f, g and h).

In the next step, each single amino acid of group 2 was investigated. The results are shown in Additional file 1: Fig. S12, S13, and S14. The OTR peak, growth rate, and lag-phases were evaluated as a criterion. Only the addition of histidine in combination with amino acid group 1 resulted in a similar peak of respiration activity as observed in the cultivation with amino acid groups 1 and 2 (Additional file 1: Fig. S12a). Growth rates were similar (Additional file 1: Fig. S13a). However, the lag phase was prolonged by 7.0 h compared to the cultivation with amino acid groups 1 and 2 (Additional file 1: Fig. S14a). The separate addition of proline and arginine resulted in a lower OTR peak value (Additional file 1: Fig. S12a), but a similar lag-phase as the cultivation supplemented with amino acid groups 1 and 2 (Additional file 1: Fig. S14a). Therefore, those two amino acids were each added to amino acid group 1 and histidine in parallel cultivations (Additional file 1: Fig. S12b). However, this did not result in entirely similar respiration activities to the cultivation with amino acid group 1 and 2 (Additional file 1: Fig. S12b, Additional file 1: Fig. S13b and Additional file 1: Fig. S14b). Additionally, a combination of amino acid group 1, histidine, proline, and arginine did not result in entirely comparable OTR courses (Additional file 1: Fig. S12c, Additional file 1: Fig. S13c and Additional file 1: Fig. S14c). Therefore, all amino acids of group 2 were used in addition to amino acid group 1 in the supplemented Moppa medium.

The studies on amino acid supplementation resulted in a better understanding of the OTR peaks. Since the small peak after around 35.3 h is only present when arginine has been added to the medium, the peak can be attributed to arginine consumption as a carbon source (Additional file 1: Fig. S12a). Additional file 1: Fig. S15 shows the good reproducibility of growth of *P. polymyxa* in three experiments performed independently of one another.

The nucleobases/-sides were reduced after reducing the amino acids to the growth-relevant ones (Additional file 1: Fig. S16). As the strain was expected to produce the nucleobases/-sides by itself, they were not divided into groups. Therefore, *P. polymyxa* was cultivated in supplemented Moppa medium containing only nicotinic acid, biotin, amino acid groups 1 and 2, with and without nucleobases/-sides. The respiration activities and offline parameters were similar for both cultivations (Additional file 1: Fig. S16). Hence, *P. polymyxa* was able to synthesize the nucleobases/-sides by itself.

The number of components in the supplemented Moppa medium was reduced from 53 to 23, similar to the number of components in the complex medium. The Moppa medium only supplemented with nicotinic acid, biotin, methionine, the amino acids of group 2 and without nucleobases/-sides is called reduced Moppa medium from now on.

### Comparability of cultivation in reduced Moppa medium in microtiter plate and laboratory fermenter

**Fig. 6:**
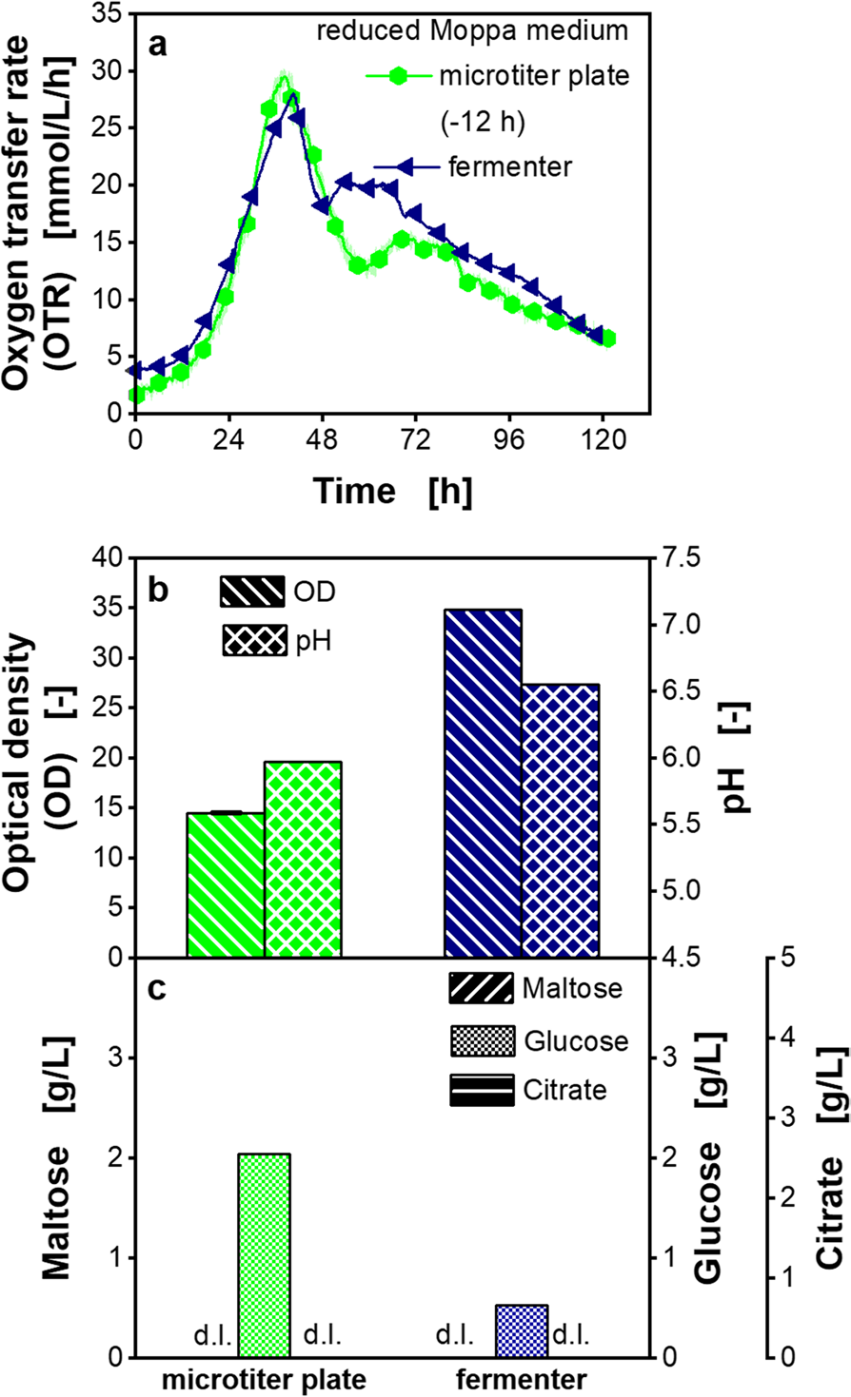
Cultivation of *Paenibacillus polymyxa* in reduced Moppa medium in microtiter plate and fermenter. Reduced Moppa medium (specified in Table 2). Initial concentrations were: 55.7–58.2 g/L maltose, 2.7–3.3 g/L glucose, 3.1–3.8 g/L citrate. **a** Oxygen transfer rate (OTR) in microtiter plate or fermenter, **b** Final optical density (OD) and pH, **c** Final maltose, glucose and citrate concentration. **c** d.l. means that concentrations of components were lower than the detection limit. Final lactate concentrations were lower than the detection limit. Parameters in b and c were determined after 133.3 h in microtiter plate (corresponds to 121.0 h cultivation time after shift) and after 119 h in fermenter. Initial osmolality in reduced Moppa medium in microtiter plate is 0.95 osmol/kg and in fermenter is 0.48 osmol/kg. Cultivation conditions: microtiter plate: temperature 33 °C, 48-round well plate, filling volume 0.8 mL, shaking frequency 1000 rpm, shaking diameter 3 mm, 0.2 M MES, initial pH 7.0. For clarity, only every 18th measuring point over time is marked as a symbol. Mean values for OTR of four replicates with standard deviations as shadows are shown for microtiter plate. Standard deviations are not well recognizable, because they are small. **b**-**c** For offline analysis, samples (wells) of the replicates of the OTR measurement were pooled at the end of the microtiter plate experiment. OD measurement of pooled sample was performed in triplicate and the mean value with standard deviation depicted as error bar is shown. pH and concentrations of sugars were determined in a single measurement of pooled sample. Fermenter: temperature 33 °C, filling volume 1 L, pH control at pH 6.5 with NH_3_ • aq and H_3_PO_4_ solutions, without MES buffer. For clarity, only every 720th measuring point is marked as a symbol. The cultivation in reduced Moppa medium in microtiter plate is also shown in Additional file 1: Figure S16

After screening optimized cultivation conditions in MTPs, the next step in bioprocess development may be the transfer to a laboratory fermenter before the final scale-up to the production scale can be performed. To validate the results of this study obtained in MTPs, a cultivation in a laboratory fermenter was performed. The respiration activities and the measured offline parameters in the MTP and the fermenter are compared in Fig. 6.

The OTR courses in both devices were comparable (Fig. 6a). The OTR of the MTP was shifted by 12 h, as growth was delayed compared to the fermenter. A higher OD was observed in the fermenter (Fig. 6b). In the fermenter, pH was controlled to a value of 6.5 by titration with 25% NH_3_ • aq and 40% H_3_PO_4_ solutions. In the MTP, the pH decreased to a value of 6.0 (Fig. 6b). The initial osmolality was two-fold higher in the cultivation in the MTP than in the fermenter (0.95 osmol/kg in MTP and 0.48 osmol/kg in fermenter). At the end of cultivation after 119 h, maltose was entirely consumed by the microorganisms in both, the fermenter and the MTP (Fig. 6c).

**Fig. 7:**
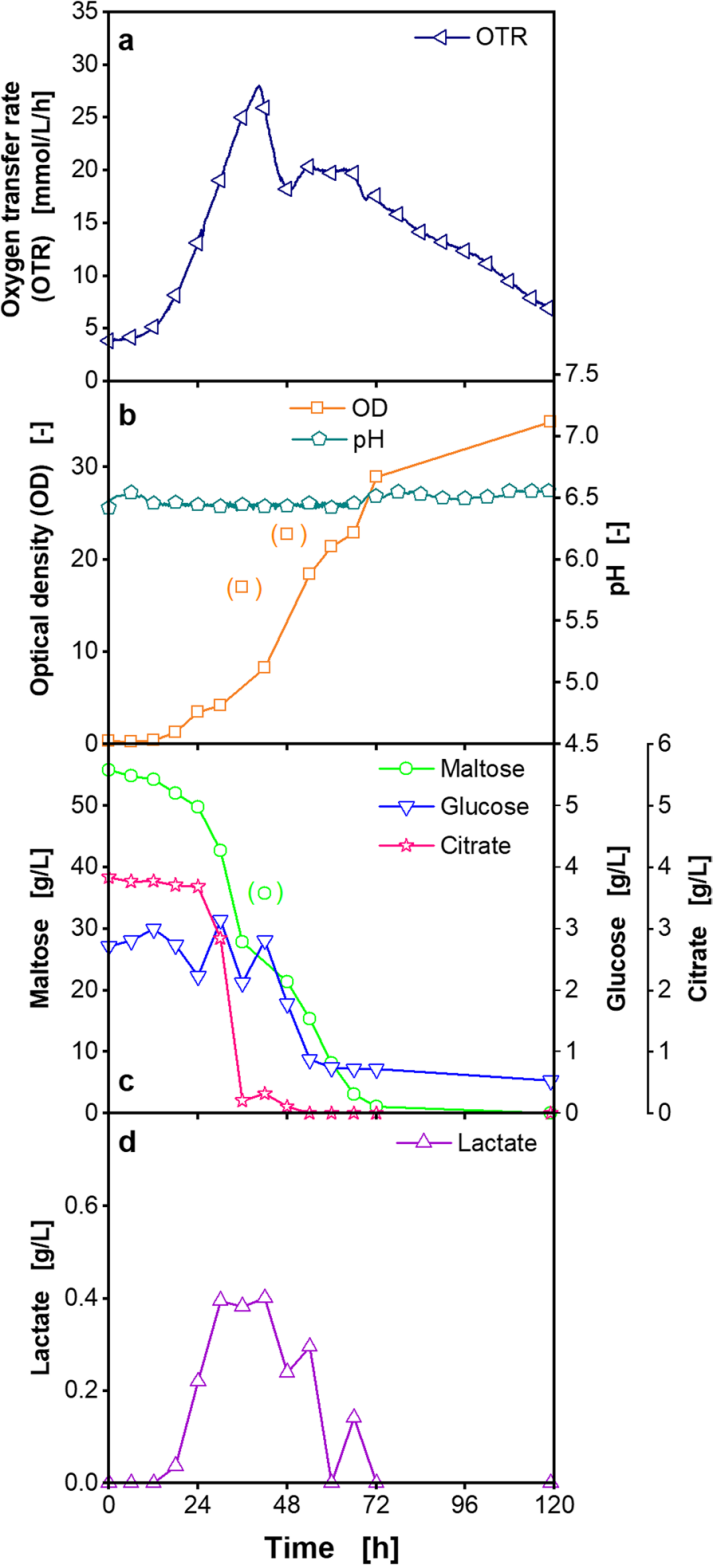
Characterization of the cultivation of *Paenibacillus polymyxa* in reduced Moppa medium in a fermenter. Reduced Moppa medium (specified in Table 2). **a** Oxygen transfer rate (OTR), **b** Optical density (OD) and pH, **c** Maltose, glucose and citrate concentration, **d** Lactate concentration. Measuring points in brackets are considered outliers. For clarity, only every 720th measuring point is marked as a symbol for OTR. Cultivation conditions: temperature 33 °C, filling volume 1 L, pH control at pH 6.5 with NH_3_ • aq and H_3_PO_4_ solutions, without MES buffer. The cultivation in the fermenter is also shown in Fig. 6

To further characterize the fermentation of *P. polymyxa*, samples were sequentially taken every 6 h over the cultivation time (Fig. 7). The OD increased throughout the whole cultivation time (Fig. 7b). When the OTR peak was reached, maltose was still available at a concentration of at least 21.3 g/L (Fig. 7c). After 119 h, maltose was completely consumed. A glucose concentration of 0.5 g/L was measured after 119 h (Fig. 7c). The citrate concentration decreased to values lower than 0.5 g/L, when the OTR peak was reached. Lactate was produced in low concentrations and then consumed in the further course of the cultivation (Fig. 7d). Lactate consumption started after the OTR peak was reached (Fig. 7d). Thereby, the assumption that lactate was consumed in cultivations with increased nicotinic acid concentrations (Fig. 2d) is confirmed here.

The dissolved oxygen (DO) dropped to 30% after 19 h and was kept constant by controlling the agitation speed in the fermenter (Additional file 1: Fig. S17). Therefore, the course of the agitation speed follows the course of the OTR curve.

## Discussion

A chemically defined medium was developed for an industrially relevant *P. polymyxa* strain, using a systematic experimental procedure, while online monitoring of the respiration activity. The medium optimization method of Müller et al. [19] was extended, to identify nutritional limitations and growth inhibitions in a chemically defined medium. In addition, the applicability of the method of Müller et al. [19] to reduce nutrient components in chemically defined media was shown. The respiration activity was used as evaluation criterion and the results were confirmed by measuring the offline parameters OD, pH, carbon source consumption, and metabolite production at the end of the cultivations.

Comparing the growth and metabolic activity of *P. polymyxa* in the initial chemically defined Moppa medium with the complex Pbp medium (Fig. 1), low respiration activities and OD values were observed in the Moppa medium. In addition, a lower consumption of carbon sources was noted in Moppa medium. After 63.3 h of cultivation in Pbp medium, no more maltose and free glucose of maltose syrup were detected. The maltose syrup mainly contains dimeric maltose molecules, maltose oligomers and free glucose. Maltose and maltose oligomers were reported to be metabolized intracellularly by *B. subtilis* [58]*.* Maltose is cleaved by a phospho-α-glucosidase after phosphorylation [59]. Maltose oligomers are hydrolyzed by a maltose phosphorylase, α-glucosidase, and neopullulanase [58]. Additionally, Hidaka et al. found a maltose phosphorylase in a *Paenibacillus* strain [60]. At 63.3 h, maltose oligomers may have remained in the complex medium. However, in this study, they were not quantified via HPLC.

The pH decreased to comparable values in the cultivations in Moppa and Pbp medium (Fig. 1). As found in *Bacillus* species [61], the decrease in pH is expected to be a result from the consumption of ammonium, which is supposed to be the major nitrogen source of the cultivation medium. Further influencing factors on the decreasing pH are acetate and lactate production, known for *P. polymyxa* [46, 62]. Acetate was not quantified via HPLC, due to overlapping peaks with MES buffer in the medium. In complex medium, no lactate was detected after 63.3 h of cultivation and a low concentration was measured in Moppa medium. Furthermore, *Paenibacillus* is well known to produce acetoin and 2,3-butanediol [46, 62]. Acetoin and butanediol concentrations were higher in Pbp medium than in Moppa medium.

Based on the comparison of the cultivations in Pbp medium and Moppa medium, nutrient limitations and inhibitions in Moppa medium were assumed. Hence, the concentrations of the supplemented nutrient groups were systematically increased in the Moppa medium. Increasing the vitamin concentrations and especially the nicotinic acid concentration by three-fold, higher OTR peaks and OD values were achieved (Additional file 1: Fig. S4a and b, Additional file 1: Fig. S5, Fig. 2a and b). Growth of *P. polymyxa* was not possible, when nicotinic acid was omitted from the cultivation medium (Fig. 2a-d). Therefore, nicotinic acid is absolutely essential for the *P. polymyxa* strain and an auxotrophy for this vitamin is proven. Nicotinic acid is the precursor for nicotinamide adenine dinucleotide (NAD +), and various genes are involved in the synthesis of NAD + [63, 64]. NAD + is the cofactor for various cellular redox reactions, for example, in the citric acid cycle [65]. Therefore, it significantly impacts bacterial biomass formation. For example in *E. coli*, nicotinic acid biosynthesis has been reported to occur via a 4-carbon dicarboxylic acid and a 3-carbon compound like glycerol [66]. The optimization of the applied *Paenibacillus* strain for industrial applications possibly led to a deletion of the relevant genes for nicotinic acid biosynthesis.

Another relevant metabolic pathway for *Paenibacillus* and *Bacillus* species is the production of acetoin and 2,3-butanediol as overflow metabolites [67]. Acetoin and 2,3-butanediol production is influenced by the NADH/NAD + availability and ratio [68]. Since pyruvate acidifies the medium, the conversion of excess pyruvate from glycolysis to acetoin and 2,3-butanediol is a mechanism to prevent strong acidification of the medium [67, 69]. In this study, the increased concentration of nicotinic acid to three-fold resulted in higher activity of metabolic pathways for biomass formation, but not in the production of higher concentrations of overflow metabolites.

Higher nicotinic acid concentrations were tested (six- and 12-fold, Fig. 2e-h). Only a plateau and a low increase of respiration activity were observed in the OTR, respectively, indicating a growth limitation by a secondary substrate or an inhibition. At the highest investigated nicotinic acid concentration, a higher biomass formation, compared to the six-fold nicotinic acid concentration, was not possible. Instead, excess pyruvate was used for overflow metabolite production.

pH optima for *P. polymyxa* strains were reported to be 6.0 or higher [49, 50]. For example, Liu. et al. showed a pH optimum for growth of 6.0 for *P. polymyxa* EJS-3 [50]. Rafigh et al. reported a pH of 7.0 optimal for biomass formation of *P. polymyxa* ATCC 21830 [49]. As the measured pH values in the present study were lower in the cultivations with higher nicotinic acid concentrations (Fig. 2f), an insufficient buffer capacity was assumed to cause the growth inhibition in the chemically defined medium. Increasing the buffer concentration and initial pH, resulted in a higher OTR peak than in the cultivation with the lowest buffer capacity (Fig. 3). This was reflected in higher OD values, but the concentration of overflow metabolites was reduced (Fig. 3b and d). This decrease can be explained by a lower activity of overflow metabolism, since pyruvate is metabolized for biomass formation in the citric acid cycle and respiratory chain, represented in the higher OD. The increase of the buffer capacity resulted in a longer lag-phase, which is traced back to the higher initial osmolality. It is well known that osmotic stress influences the growth of microorganisms [70, 71] and can prolong the lag-phase [3].

As higher respiration activities in supplemented Moppa medium were achieved than in complex medium, the next step was to reduce the medium components to the essential and growth-relevant ones. The vitamins were reduced to nicotinic acid and biotin (Fig. 4). As growth was possible without biotin, but a longer cultivation time was required (Additional file 1: Fig. S10), biotin was identified as a growth-enhancing vitamin, which, however, is not absolutely essential for the *P. polymyxa* strain. For amino acids, an auxotrophy for methionine was shown, as growth of *P. polymyxa* was not possible without methionine (Fig. 5a-d). In addition, a combination of histidine, arginine, proline, and glutamate enhanced growth (Fig. 5a-d). Omitting nucleobases/ -sides did not influence the growth of *P. polymyxa* (Additional file 1: Fig. S16) and can, therefore, be left out of the medium. After reduction of the medium components to the essential and growth-relevant ones, a comparable number of media components (23) in the optimized chemically defined medium, the reduced Moppa medium, as in the Pbp complex medium (22) was achieved.

During reduction of medium components, it was observed that the OTR peak in the cultivation supplemented only with amino acid group 1 was lower than the OTR peak in the cultivation supplemented with all amino acids (Fig. 5a). The TOC was at comparable levels. However, the OD was higher in the cultivation supplemented only with amino acid group 1 (Fig. 5b). The reason for the higher OD is not yet clear. One possible explanation is an optical interference due to morphological changes. Those morphological changes might be caused by cell lysis due to sporulation of *P. polymyxa*. It is known that amino acids influence the sporulation of *Bacillus* species [72].

Finally, the cultivation of *P. polymyxa* in the chemically defined medium was successfully reproduced in a laboratory fermenter (Fig. 6). Hence, the results showed a good transferability of the developed medium, based on online monitoring, although the method of pH control was different. The larger lag-phase in MTP might be driven by the higher initial osmolality in the cultivation in the MTP. Based on internal data (not published), increasing osmotic pressures (higher than 0.80 osmol/kg) were found to incrementally reduce growth of the *P. polymyxa* strain. The higher osmolality results from the used buffer in the MTP cultivation. In contrast, the pH in the fermenter was controlled by titration. A higher OD was achieved in the fermentation than in the cultivation in the MTP (Fig. 6b). This is likely the influence of the different pH control strategies. The reasons for the differences in OD have to be investigated in further studies.

## Conclusions

In this study, a chemically defined medium for a *P. polymyxa* strain was developed by combining a systematic experimental procedure and the small-scale high-throughput online cultivation system µRAMOS. In comparison to complex Pbp medium, the respiration activity and optical density in the chemically defined Moppa medium were low. Nicotinic acid was identified as a growth-limiting component. Furthermore, the use of the online monitoring technique revealed a pH inhibition in the small-scale cultivations, which was traced back to an insufficient buffer capacity. By increasing the nicotinic acid concentration, the initial pH and the buffer concentration in the Moppa medium, similar respiration activities to the Pbp medium were achieved. The media components in the supplemented Moppa medium were reduced to a comparable number (23) as the components in Pbp medium (22). Auxotrophies for nicotinic acid and methionine were shown for the applied *P. polymyxa* strain. Biotin and the combination of histidine, arginine, proline, and glutamate showed growth-enhancing effects. Nucleobases /-sides do not have to be added to the medium. The information obtained from the online monitored respiration activities in the experiments could be verified by offline measured parameters (OD, pH, carbon source consumption, and metabolite production).

This study shows that online monitoring of respiration activity in high-throughput MTP cultivations in combination with a systematic experimental procedure is a valuable tool to optimize cultivation media. Compared to conventional methods, like OFAT, the number of experiments was reduced. A screening procedure was established for *P. polymyxa* in MTPs. Finally, the good transferability of the MTP results to a laboratory fermenter highlights the importance of online monitoring the respiration activity in small-scale cultivation systems.

## Methods

### Microbial strain

The cultivations were performed with a *Paenibacillus polymyxa* strain, which was obtained from BASF SE (Ludwigshafen am Rhein, Germany). This strain was generated by random-mutagenesis of a wild-type isolate, in order to abolish the production of the human last-line antibiotic polymyxin, without affecting growth and sporulation. The strain was designated as *P. polymyxa* JG1*.* Further information is available from BASF SE (Ludwigshafen am Rhein, Germany) on request.

### Cultivation media

A complex medium was used for pre-cultures. The composition of the medium is listed in Table 1. The trace element, vitamin, and Ca(NO_3_)_2_ stock solutions were sterilized by filtration (0.2 µm). The maltose stock solution was autoclaved at 121 °C for 60 min. The pH of the stock solution containing citric acid, K_2_HPO_4_, (NH_4_)_2_SO_4_, MgSO_4_, yeast extract (Bio Springer, Maisons-Alfort, France), and soy flour (Sofarine Bic Protein, BiC, BC’s-Hertogenbosch, The Netherlands) was checked to be lower than 6.0. Then antifoam (Struktol J673, Schill + Silacher “Struktol” GmbH, Hamburg, Germany) was added and the solution was autoclaved. Immediately before starting the cultivation, all stock solutions were combined, and the pH of the medium was adjusted to 6.5 using 25% (w/w) NH_3_ • aq and 40% (w/w) H_3_PO_4_ solutions.

Main-cultures were performed in complex or in chemically defined medium. The complex medium was the same as for pre-cultures with some modifications and was here called *Paenibacillus polymyxa* (Pbp) complex medium (Table 1). This Pbp medium contained 120 g/L glucose syrup (C✩Sweet™ M, Cargill Deutschland GmbH, Krefeld, Germany) instead of 60 g/L maltose and additionally 0.1 M 2-(*N*-morpholino)ethanesulfonic acid (MES) buffer. In the study presented herein, the glucose syrup is called maltose syrup. It contains around 50% (w/w) dimeric maltose molecules, 21% (w/w) maltose oligomers, and 2% (w/w) glucose, resulting in an initial dimeric maltose concentration of around 60 g/L in the Pbp complex medium. The pH of the MES buffer stock solution was adjusted to 6.5 with 50% (w/v) NaOH. Maltose syrup stock solution was autoclaved, and MES buffer was sterile filtered (0.2 µm). Preparation of the medium was performed as described for the pre-culture complex medium.

The chemically defined medium for main-cultures is called Moppa medium. This medium is based on a chemically defined medium after Müller et al. [19] and was modified. The composition of the Moppa medium and its variations are listed in Table 2. The maltose syrup, citric acid/ (NH_4_)_2_SO_4_, and antifoam stock solutions were autoclaved. All other stock solutions were sterile filtered. The MES buffer was prepared as for the complex medium, and the K_2_HPO_4_ solution was adjusted to a pH of 6.5 with 40% (w/w) H_3_PO_4_ solution. The pH of individual amino acid, vitamin and nucleobases/-sides stock solutions was changed with KOH or HCl solutions if they were not water soluble in the required concentration. The iron stock solution was frozen in aliquots at -20 °C after sterile filtration. Immediately before starting the cultivation, all medium stock solutions were combined except K_2_HPO_4_, and the pH was adjusted to 6.5 with 25% (w/w) NH_3_ • aq solution and 40% (w/w) H_3_PO_4_ solution. After that, the K_2_HPO_4_ solution was added. The medium composition was adjusted for each experiment shown in the respective figures, and the preparation of stock solutions was adapted for the investigated components. For the fermentation the reduced Moppa medium, specified in Table 2, was prepared with 0.4 g/L D/L-methionine instead of 0.2 g/L L-methionine.

### Cultivation conditions

For pre-cultures, 30 mL medium was inoculated with 180 µL of cryo-culture in a baffled 250 mL shake flask closed with a plug (Silicosen®, Hirschmann Laborgeräte GmbH & Co. KG, Eberstadt, Germany). Cultivations were performed at 33 °C with a shaking diameter of 25 mm, and a shaking frequency of 150 rpm for 24–26 h.

Main cultures were inoculated with 2.0% (v/v) from pre-culture for the microtiter plates (MTP) and fermenter cultivations. The main-cultures in MTPs were conducted in 48-round well MTPs without optodes (MTP-R48-B, Beckman Coulter GmbH, Krefeld, Germany) at 33 °C with a shaking diameter of 3 mm, a shaking frequency of 1000 rpm, and a filling volume of 0.8 mL in complex Pbp medium or chemically defined Moppa medium. MTPs were closed with a gas-permeable sealing foil (900,371-T, HJ-Bioanalytik GmbH, Erkelenz, Germany). The in-house Respiration Activity MOnitoring System in MTPs (µRAMOS) was used for online monitoring of the oxygen transfer rate (OTR) [40].

The fermentation was performed in a stirred tank reactor (1.8 L Benchtop fermentation system, DasGip, Eppendorf, Hamburg, Germany). The cultivation was conducted in batch mode with a liquid volume of 1 L at 33 °C. The dissolved oxygen (DO) was controlled at 30% with a stirrer-gas flow cascade (400 to 1400 rpm for a DO of 0 to 40% and 18–180 sL/h for a DO of 40 to 100%). The pH was controlled at 6.5 using 25% NH_3_ • aq and 40% H_3_PO_4_ solutions. BlueSens sensors (Herten, Germany) were used for exhaust gas analytics. Antifoam addition (Struktol J673, Schill + Seilacher “Struktol” GmbH, Hamburg, Germany) was performed on demand by using an antifoam probe.

### Offline analysis

For offline analysis, samples of at least three replicates of the wells from the MTP were pooled at the end of the experiments to increase sample volume to at least 2.0 mL. The optical density (OD) was measured at a wavelength of 600 nm with a Genesys 20 photometer (Thermo Scientific, Darmstadt, Germany) or a Lambda photometer Bio + (PerkinElmer, Rodgau, Germany). Samples were diluted with 0.9% (w/v) NaCl to stay in the linear range of 0.1 to 0.3. The pH of the culture broth was measured using a HI221 Basic pH (Hanna Instruments Deutschland GmbH, Vöhringen, Germany) or pH-Meter 766 Calimatic (Knick, Berlin, Germany). For osmolality and high-performance liquid chromatography (HPLC) of sugars and metabolites, samples were centrifuged for 5 min at 13000 g, and the supernatant was filtrated (0.2 µm). The osmolality of filtrated supernatant was determined with a freezing point Osmomat 3000 basic (Gonotec GmbH, Berlin, Germany) or an Advanced OsmoPro (Advanced Instruments, Norwood, MA, USA). For HPLC, samples were diluted with deionized water. Sample analysis for MTP experiments was performed with a Dionex UltiMate 3000 HPLC system (Thermo Scientific, Darmstadt, Germany). Measurements were conducted at 80 °C with 5 mM sulfuric acid as solvent with a flow of 0.8 mL/min and two connected organic acid-resin columns (300*7.8 mm, Phenomenex, Aschaffenburg, Germany). Refractive index (RI) measurement was performed for all components with a RI detector (RefractoMax 520, Shodex, Munich, Germany). Citrate was determined via UV signal with a Dionex UltiMate 3000 Diode Array Detector (Thermo Scientific, Darmstadt, Germany) or RI detector. HPLC analyses of fermentation samples were conducted with an Agilent HPLC system (Agilent, Santa Clara, CA, USA) at 30 °C using 5 mM sulfuric acid as solvent with a flow of 0.5 mL/min and an Aminex HPX-87 H column (300*7.8 mm, Bio-Rad Laboratories, Hercules, CA, USA). All components were detected via RI-detector (G1362A from Agilent, Santa Clara, CA, USA). A comparative measurement of the two described methods above was performed, and the values in the fermenter were converted with a correlation (data not shown). Samples from the beginning of each experiment were analyzed by HPLC. The concentration ranges of those initial samples are specified in the corresponding Figure captions. For cultivations in MTPs, evaporation was considered for OD and HPLC measurement by weighing the MTP filled with culture broth at the beginning and end of the cultivation.

### Determination of lag-phase

The lag-phase was determined based on a method after Palmen et al. [73], with some modifications. The logarithm of the difference between the OTR and initial OTR was calculated and plotted over time. The initial OTR was determined as a mean value of OTR values of a cultivation time between 1.6 to 3.0 h. This procedure was used to average low fluctuations of the initial measurement points. To specify the lag-phase, the intersection point of the regression line of the linear range was determined.

### Depiction of experimental results of microtiter plate experiments

In MTP cultivations three to six replicates were performed, depending on the experiment. Mean values of the OTR are shown in the corresponding figures. Due to the very high density of the data of the OTR, not every measurement point over time could be represented as a symbol. Therefore, to clearly display the data, the OTR is only shown for a portion of the measured values as a symbol. The number of shown measuring points depends on the cultivation time of the experiment and is indicated in the respective figure caption. The standard deviation of the OTR data in MTP experiments is displayed as shadows. The TOC was determined based on the OTR of the replicates. The TOC is depicted as mean values of the replicates with error bars in bar charts. For offline analysis, samples (wells) of the replicates of the MTP cultivations were pooled at the end of the experiments. The results of measurements of pooled OD, pH, and sugar and metabolite concentrations of MPT experiments are displayed in bar charts. The OD of the pooled sample was measured in triplicate, represented as mean value with standard deviation as error bar. The pH and HPLC measurement of the pooled sample were performed in single determination and the single values are shown in bar charts.

### Statistical analysis

Statistical analysis was performed using R (version 4.2.2, R Core Team, 2022, R Foundation for Statistical Computing, Vienna, Austria) with RStudio (version 2022.12.0, Posit team, 2022, Posit Software, PBC, Boston, MA, USA) as integrated development environment. To investigate statistical significance of differences of OTR peaks, a t-test (equal variances, two-sided) was conducted, when two samples were compared. An ANOVA followed by a Bonferroni post-hoc test was used, when more than two samples were investigated. A p-value of lower than 0.05 was determined to show significance in all statistical tests.

## Supplementary Information

**Additional file 1. **

## Data Availability

The data that support the findings of this study are available from BASF SE, but restrictions apply to the availability of these data, which were used under license for the current study, and so are not publicly available. Data are however available from the corresponding author upon reasonable request and with permission of BASF SE.

## Abbreviations

CTR: Carbon dioxide transfer rate
DO: Dissolved oxygen
gr.: Group
HPLC: High-performance liquid chromatography
MES: 2-(*N*-Morpholino)ethanesulfonic acid
MTP: Microtiter plate
Moppa medium: Chemically defined medium for *Paenibacillus polymyxa*
NAD +: Nicotinamide adenine dinucleotide
nic.: Nicotinic
OD: Optical density
OFAT: One-factor-at-a-time method
OTR: Oxygen transfer rate
pantoth.: Pantothenic
p-benz.: p-Aminobenzoic
Pbp complex medium: *Paenibacillus polymyxa* complex medium
pH_0_: Initial pH
RAMOS: Respiration Activity MOnitoring System for shake flasks
RI: Refractive index
RQ: Respiratory quotient
µRAMOS: Respiration Activity MOnitoring System for microtiter plates
TOC: Total oxygen consumption
