# Supplementary material for: Development of a chemically defined medium for Paenibacillus polymyxa by parallel online monitoring of the respiration activity in microtiter plates
Source: BMC Biotechnol. 2023 Jul 28;23:25. doi: 10.1186/s12896-023-00793-7 (PMC10385886; doi:10.1186/s12896-023-00793-7)
Supplement: Supplementary file 1 — Additional file 1. [file 12896_2023_793_MOESM1_ESM.pdf]

Additional file 1

Table S1: Statistically significance analysis of OTR peaks of cultivation in complex and chemically defined medium. The statistical results correspond to the cultivations shown in Figure 1. A t-test (equal variances, two-sided) was performed. A p-value < 0.05 was determined to show statistical significance.

	**Pbp complex medium**	**Moppa medium**
p-value	<0.001	

Table S2: Groups of amino acids in Moppa medium.

Group #	1	2	3	4	5	6
Amino acids	Methionine	Histidine	Aspartate	Cysteine	Phenylalanine	Alanine
		Proline	Isoleucine	Serine	Tyrosine	Leucine
		Glutamate	Threonine	Glycine	Tryptophane	Lysine
		Arginine				Valine

Table S3: Groups of vitamins in Moppa medium.

Group #	1	2	3	4
Vitamins	Nicotinic acid	Folic acid	Riboflavin	Biotin
	Pantothenic acid	Ascorbic acid	Orotic acid	
	p-Amino-benzoic acid		Inositol	
	Pyridoxamine			
	Pyridoxine			
	Thiamine			
	Vitamin B12			

Table S4: Statistically significance analysis of OTR peaks of cultivation with increased concentration of nicotinic acid. gr.: group, nic.: nicotinic. The statistical results correspond to the cultivations shown in Figure 2a. An ANOVA followed by a Bonferroni post-hoc test was performed. A p-value < 0.05 was determined to show statistical significance. The p-value of ANOVA was <0.001.

	**Moppa medium**	**Moppa medium**	
		**w/ 3x vitamin gr. 1**	**w/ 3x nic. acid**
**Moppa medium**	-	-	-
**w/ 3x vitamin gr. 1**	p-value <0.001	-	-
**w/ 3x nic. acid**	p-value <0.001	p-value = 0.012	-

Table S5: Statistically significance analysis of OTR peaks of cultivations with increased pH – buffer capacity. nic.: nicotinic. The statistical results correspond to the cultivations shown in Figure 3. An ANOVA followed by a Bonferroni post-hoc test was performed. A p-value < 0.05 was determined to show statistical significance. The p-value of ANOVA was <0.001.

	**Moppa medium w/ 12x nic. acid**		
	**0.1 M MES, pH_0=6.5**	**0.2 M MES, pH_0=6.5**	**0.2 M MES, pH_0=7.0**
**0.1 M MES, pH_0=6.5**	-	-	-
**0.2 M MES, pH_0=6.5**	p-value = 0.009	-	-
**0.2 M MES, pH_0=7.0**	p-value <0.001	p-value <0.001	-

Table S6: Statistically significance analysis of OTR peaks of cultivation only with growth relevant vitamins. nic.: nicotinic. The statistical results correspond to the cultivations shown in Figure 4. A t-test (equal variances, two-sided) was performed. A p-value < 0.05 was determined to show statistical significance.

	**Supplemented Moppa medium**	
	**w/ vitamins**	**w/o vitamins, only w/ 12x nic. acid, 1x biotin**
p-value	0.137	

Table S7: Statistically significance analysis of OTR peaks of cultivation with varying amino acid composition. gr.: group, nic.: nicotinic. The statistical results correspond to the cultivations shown in Figure 5. An ANOVA followed by a Bonferroni post-hoc test was performed. A p-value < 0.05 was determined to show statistical significance. The p-value of ANOVA was <0.001.

	**Supplemented Moppa medium, w/o vitamins, only w/ 12x nic. acid, 1x biotin**						
	**w/ all amino acids**	**w/ amino acid gr. 1**	**w/ amino acid gr. 1, 2**	**w/ amino acid gr. 1, 3**	**w/ amino acid gr. 1, 4**	**w/ amino acid gr. 1, 5**	**w/ amino acid gr. 1, 6**
**w/ all amino acids**	-	-	-	-	-	-	-
**w/ amino acid gr. 1**	<0.001	-	-	-	-	-	-
**w/ amino acid gr. 1, 2**	1.000	<0.001	-	-	-	-	-
**w/ amino acid gr. 1, 3**	<0.001	1.000	<0.001	-	-	-	-
**w/ amino acid gr. 1, 4**	<0.001	0.003	<0.001	<0.001	-	-	-
**w/ amino acid gr. 1, 5**	<0.001	1.000	<0.001	1.000	0.002	-	-
**w/ amino acid gr. 1, 6**	<0.001	1.000	<0.001	0.750	0.007	1.000	-

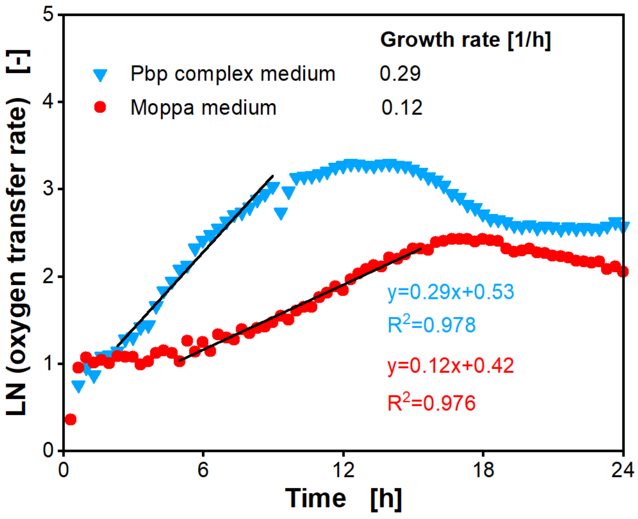

Figure S1: Determination of growth rate in complex and chemically defined Moppa medium. Pbp complex medium (specified in Table 1) or chemically defined Moppa medium (specified in Table 2). Growth rates of *Paenibacillus polymyxa* are calculated based on regression of the linear range of logarithm of oxygen transfer rate (OTR). OTRs are shown in Figure 1a. Cultivation conditions: temperature 33 °C, 48-round well plate, filling volume 0.8 mL, shaking frequency 1000 rpm, shaking diameter 3 mm, 0.1 M MES, initial pH 6.5.

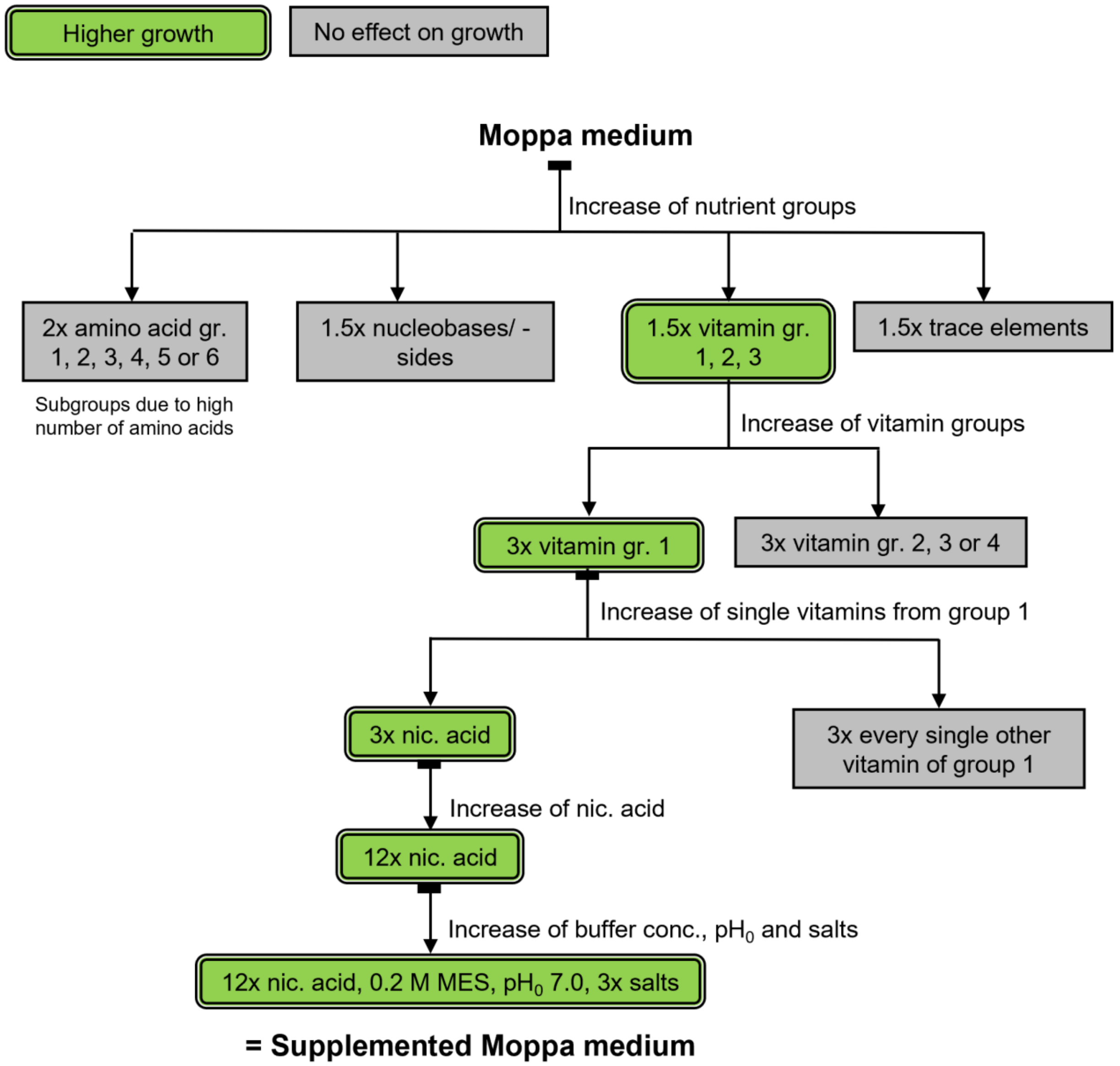

Figure S2: Schematic overview for systematic identification of growth limitations in Moppa medium for *Paenibacillus polymyxa*. Compositions of Moppa medium and supplemented Moppa medium are specified in Table 2. Amino acid and vitamin groups are specified in Additional file 1: Table S2 and S3, respectively. nic.: nicotinic, pH_0: initial pH, salts: $(NH_4)_2SO_4$, K_2HPO_4. Green boxes with double lined edging: growth and metabolic activity (Optical density and oxygen transfer rate peak or total oxygen consumed) are higher than in the reference cultivation. Grey boxes with single line edging: growth and metabolic activity are comparable to the reference cultivation. Bar at the end of the arrow marks the used medium reference, until a new arrow with bar is shown.

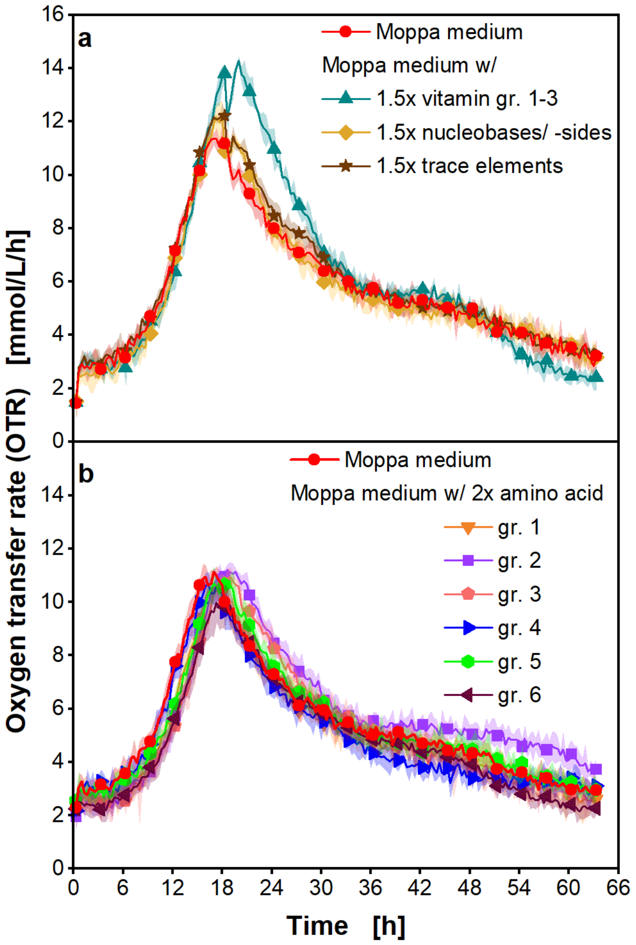

Figure S3: Cultivation of *Paenibacillus polymyxa* with increased concentrations of nutrient groups in microtiter plate. Moppa medium (specified in Table 2) or Moppa medium with 1.5-fold concentration of vitamin group 1, 2 or 3 specified in Additional file 1: Table S3, nucleobases/-sides or trace elements or Moppa medium with 2-fold concentration of amino acid groups specified in Additional file 1: Table S2. a, b: Oxygen transfer rate (OTR). For clarity, only every 10th measuring point over time is marked as a symbol. Mean values for OTR of at least four replicates with standard deviations as shadows are shown. Cultivation conditions: Temperature 33 °C, 48-round well plate, filling volume 0.8 mL, shaking frequency 1000 rpm, shaking diameter 3 mm, 0.1 M MES, initial pH 6.5.

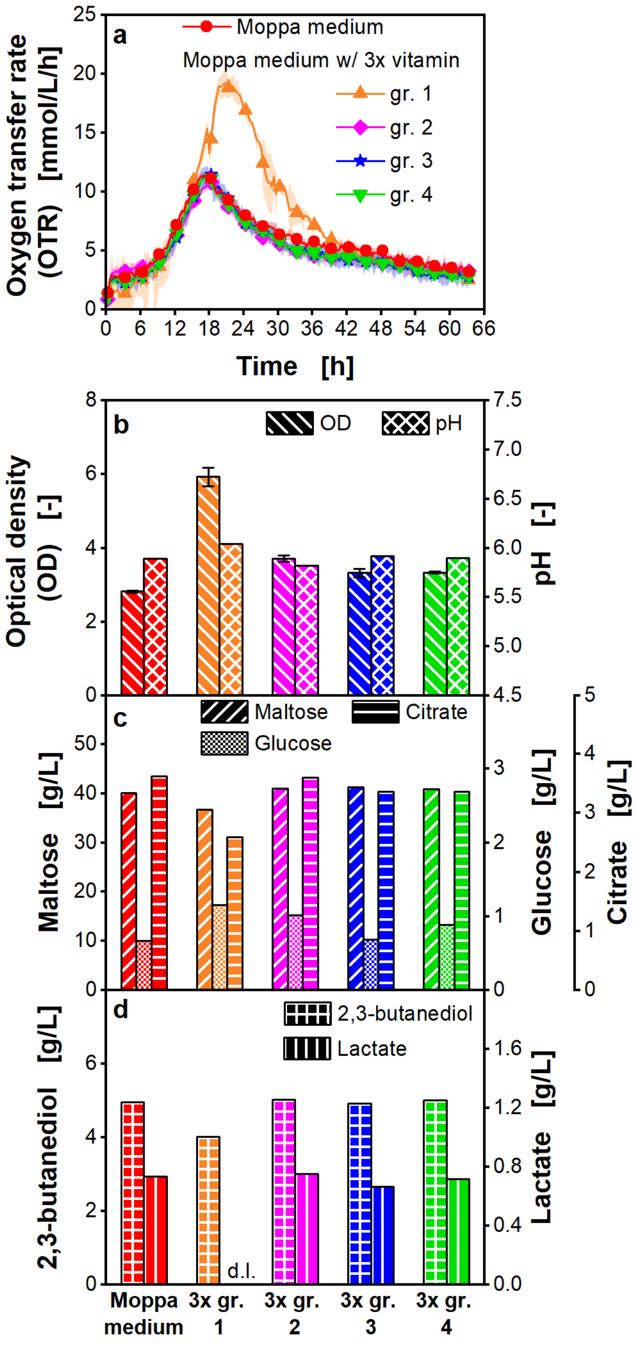

Figure S4: Cultivation of *Paenibacillus polymyxa* with increased concentrations of vitamin groups in microtiter plate. Moppa medium (specified in Table 2) or Moppa medium with increased concentrations of vitamin groups. Vitamin groups (gr.) are specified in Additional file 1: Table S3. Initial concentrations were: 55.0-56.7 g/L maltose, 3.5 g/L glucose, 3.0-3.1 g/L citrate. a: Oxygen transfer rate (OTR), b: Final optical density (OD) and pH, c: Final maltose, glucose and citrate concentration, d: Final 2,3-butanediol and lactate concentration. a: For clarity, only every 10th measuring point over time is marked as a symbol. Mean values for OTR of at least four replicates with standard deviations as shadows are shown. b-d: For offline analysis, samples (wells) of the replicates of the OTR measurement were pooled at the end of the experiments. OD measurement of pooled samples was performed in triplicate and mean values with standard deviations depicted as error bars are shown. pH and concentrations of sugars and metabolites were determined in a single measurement of pooled samples. d: d.l. means that concentrations of components were lower than the detection

limit. Final acetoin concentrations were lower than the detection limit. Parameters in b-d were determined after 63.3 h. Cultivation conditions: temperature 33 °C, 48-round well plate, filling volume 0.8 mL, shaking frequency 1000 rpm, shaking diameter 3 mm, 0.1 M MES, initial pH 6.5.

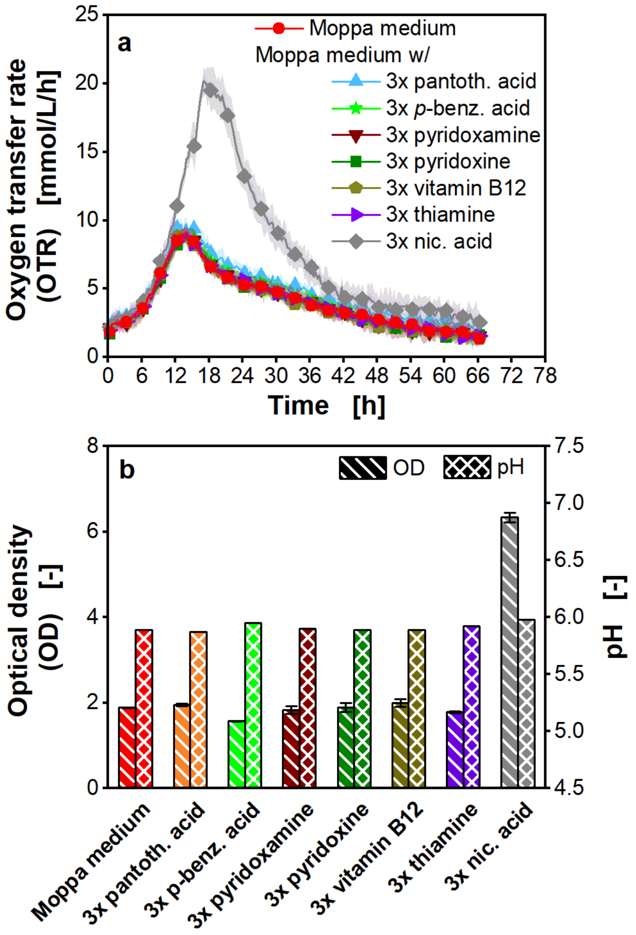

Figure S5: Cultivation of *Paenibacillus polymyxa* with increased concentrations of vitamins of group 1 in microtiter plate. Moppa medium (specified in Table 2) or Moppa medium with increased concentrations of vitamins of group 1 specified in Additional file 1: Table S3. Pantoth.: pantothenic, *p*-benz.: *p*-aminobenzoic, nic.: nicotinic. a: Oxygen transfer rate (OTR), b: Final optical density (OD) and pH. a: For clarity, only every 10th measuring point over time is marked as a symbol. Mean values for OTR of at least three replicates with standard deviations as shadows are shown. b: For offline analysis, samples (wells) of the replicates of the OTR measurement were pooled at the end of the experiments. OD measurement of pooled samples was performed in triplicate and mean values with standard deviations depicted as error bars are shown. pH was determined in a single measurement of pooled samples. Optical density and pH in b were determined after 66.3 h. The cultivation with the three-fold nicotinic acid concentration is shown in more detail in Figure 2a-d. Cultivation conditions: temperature 33 °C, 48-round well plate, filling volume 0.8 mL, shaking frequency 1000 rpm, shaking diameter 3 mm, 0.1 M MES, initial pH 6.5.

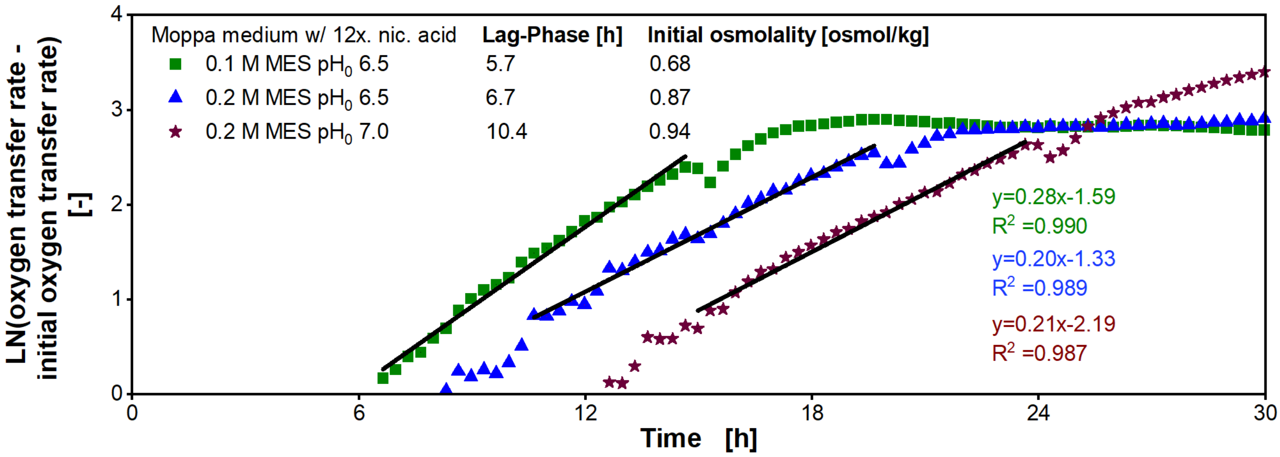

Figure S6: Determination of lag-phase for *Paenibacillus polymyxa* with increased pH - buffer capacity. Moppa medium (specified in Table 2) with 12x nicotinic acid or Moppa medium with 12x nicotinic acid and with increased buffer concentration or with increased buffer concentration and initial pH. nic.: nicotinic, pH_0: initial pH. The logarithm of the difference of the oxygen transfer rate (OTR) and initial OTR was plotted over time. The initial OTR was determined based on the mean value of OTR values between 1.6 and 3.0 h. The lag-phases are calculated based on the intersection point of the regression line of the linear range. OTRs are shown in Figure 3a. Osmolality after 86.3 h in Moppa medium with 12x nicotinic acid, 0.1 M MES and initial pH 6.5 is 0.64 osmol/kg. Osmolality after 86.3 h in Moppa medium with 0.2 M MES and initial pH 6.5 is 0.71 osmol/kg. Osmolality after 86.3 h in Moppa medium with 0.2 M MES and initial pH 7.0 is 0.73 osmol/kg. Cultivation conditions: temperature 33 °C, 48-round well plate, filling volume 0.8 mL, shaking frequency 1000 rpm, shaking diameter 3 mm.

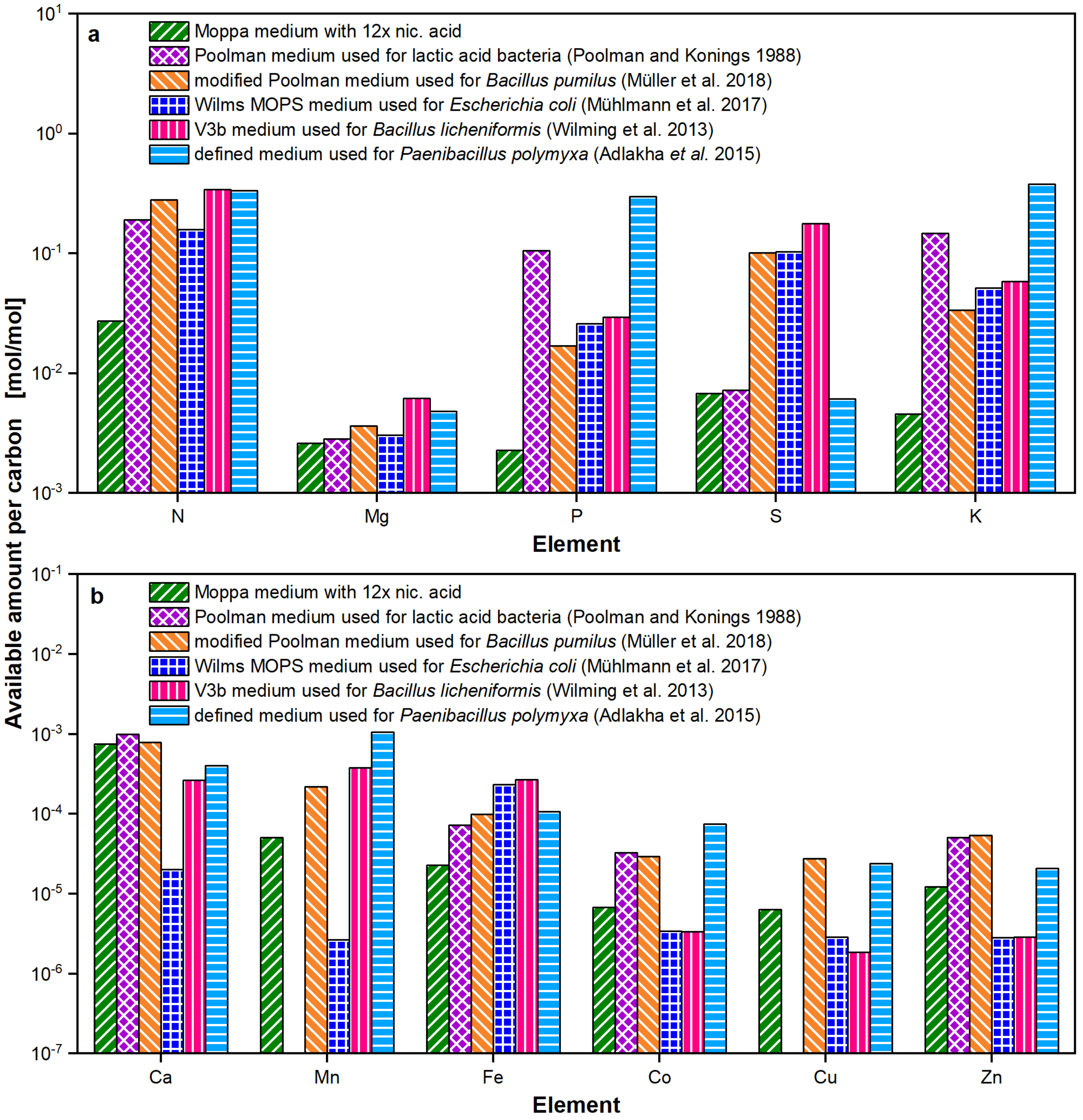

Figure S7: Elemental composition of Moppa medium, compared to established chemically defined media. Moppa medium (specified in Table 2) with 12x nicotinic acid and literature known media for various bacteria [19, 30, 31, 51, 57]. a: Amounts are calculated in amount of nitrogen (N), magnesium (Mg), phosphor (P), sulfur (S), and potassium (K) per amount of carbon. b: Amounts are calculated in amount of calcium (Ca), manganese (Mn), iron (Fe), cobalt (Co), copper (Cu), and zinc (Zn) per amount of carbon. For calculation of the amount of carbon (C) in Moppa medium with 12x nicotinic acid, 60 g/L maltose, 2.6 g/L glucose, 3.6 g/L citrate, and carbon of amino acids, vitamins and nucleobases/-sides were considered. In all other media containing amino acids, vitamins and nucleobases/-sides, the carbon of those nutrients was also considered. For calculation of the amount of N, Mg, P, S, K, Ca, Mn, Fe, Co, Cu, Zn, not only the amount of those elements in the salts and trace elements was considered, but also the amount of those elements in other medium components, like vitamins, amino acids and nucleobases/-sides. Y-axis: logarithmic scale.

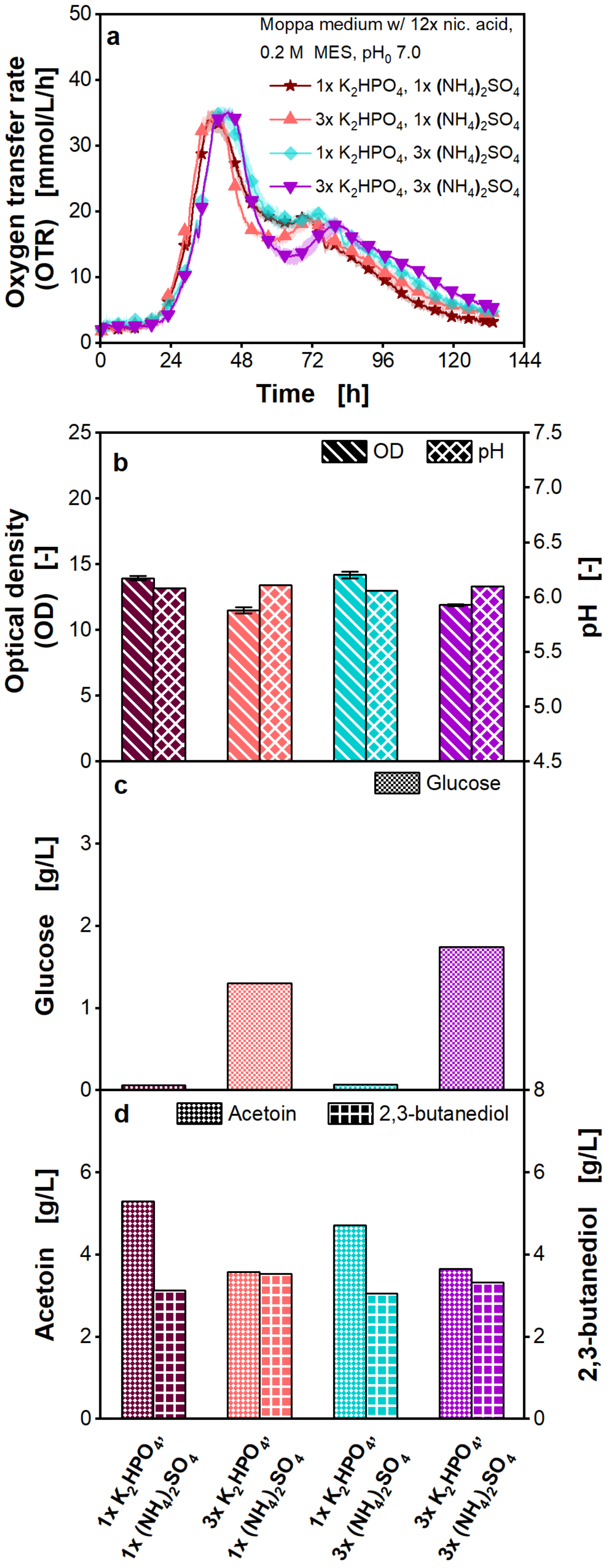

Figure S8: Cultivation of *Paenibacillus polymyxa* with increased concentration of salts in microtiter plate. Moppa medium (specified in Table 2) with 12x nicotinic acid, 0.2 M MES, initial pH 7.0 or Moppa medium with 12x nicotinic acid, 0.2 M MES, initial pH 7.0 and with increased concentration of K_2HPO_4 and/

or $(NH_4)_2SO_4$. nic.: nicotinic, pH_0: initial pH. Initial concentrations were: 57.0-57.4 g/L maltose, 3.1-3.4 g/L glucose, 3.0-3.2 g/L citrate. a: Oxygen transfer rate (OTR), b: Final optical density (OD) and pH, c: Final glucose concentration, d: Final acetoin and 2,3-butanediol concentration. a: For clarity, only every 18th measuring point over time is marked as a symbol. Mean values for OTR of at least four replicates with standard deviations as shadows are shown. b-d: For offline analysis, samples (wells) of the replicates of the OTR measurement were pooled at the end of the experiments. OD measurement of pooled samples was performed in triplicate and mean values with standard deviations depicted as error bars are shown. pH and concentrations of sugars and metabolites were determined in a single measurement of pooled samples. Final maltose, citrate and lactate concentrations were lower than the detection limit. Parameters in c-d were determined after 133.3 h. Cultivation conditions: temperature 33 °C, 48-round well plate, filling volume 0.8 mL, shaking frequency 1000 rpm, shaking diameter 3 mm. The medium with 3x K_2HPO_4 und 3x $(NH_4)_2SO_4$ is called supplemented Moppa medium.

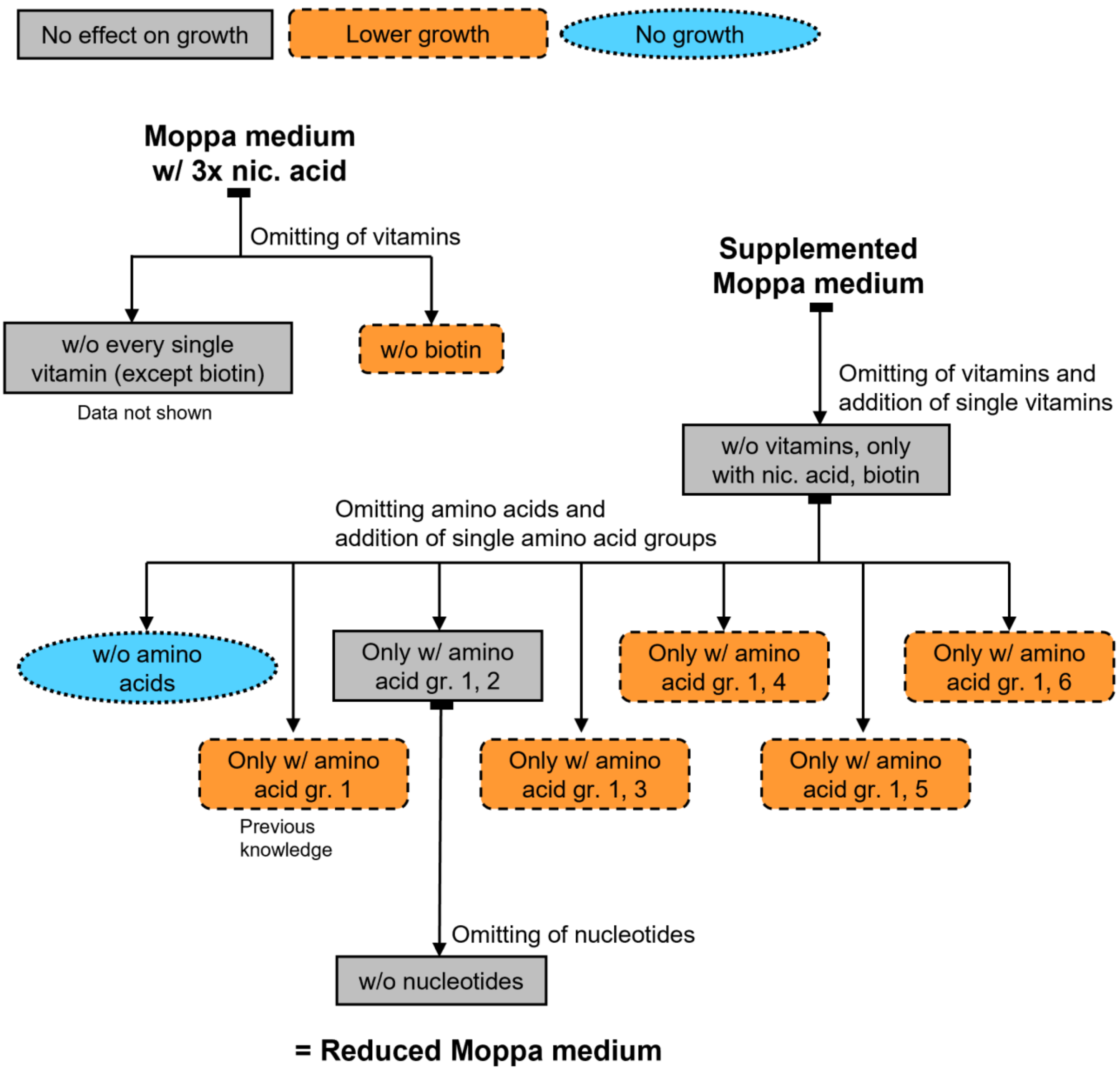

Figure S9: Schematic overview for systematic reduction of medium ingredients in supplemented Moppa medium for *Paenibacillus polymyxa*. Compositions of Moppa medium, supplemented Moppa medium and reduced Moppa medium are specified in Table 2. Amino acid and vitamin groups are specified in Additional file 1: Table S2 and S3, respectively. nic. acid: nicotinic acid. Grey boxes with solid single line edging: growth and metabolic activity (optical density and oxygen transfer rate peak or total oxygen consumed) is comparable to the reference cultivation. Light brown boxes with dashed line edging: Growth and metabolic activity is lower than in the reference cultivation. Blue ellipse with dotted line edging: No growth is observed. Bar at the end of the arrow marks the used medium reference until a new arrow with bar is shown.

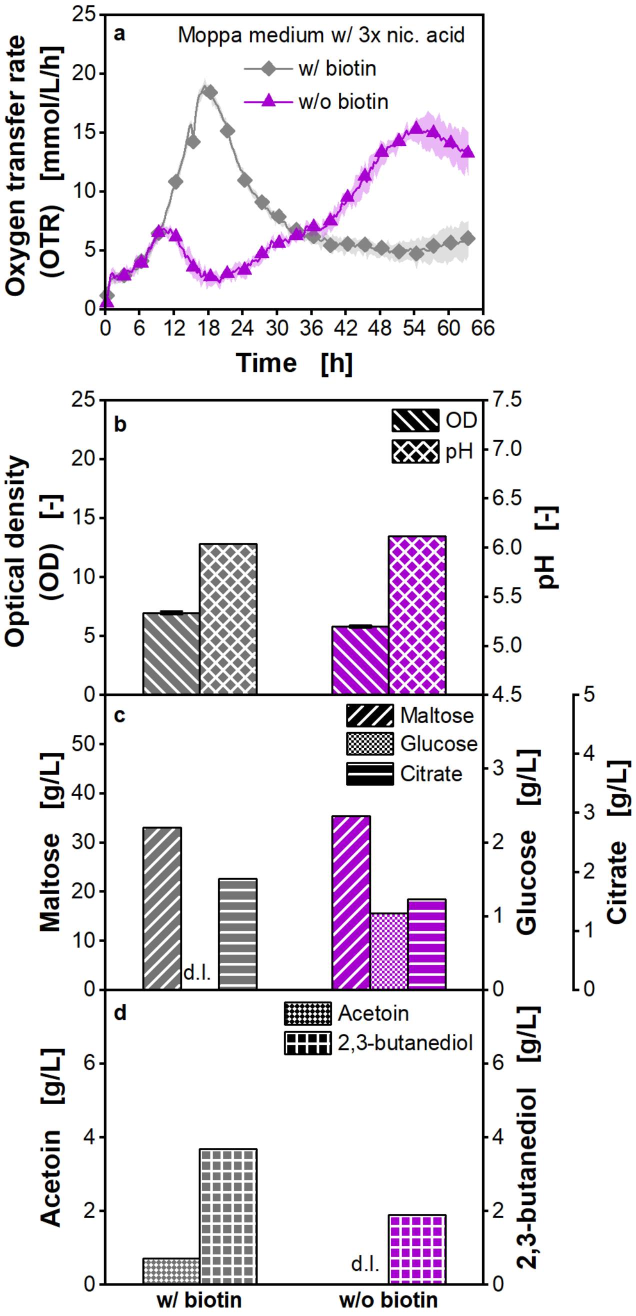

Figure S10: Cultivation of *Paenibacillus polymyxa* without biotin in microtiter plate. Moppa medium (specified in Table 2) with 3x nicotinic acid with and without biotin. nic.: nicotinic. Initial concentrations were: 53.0-53.1 g/L maltose, 4.2-4.4 g/L glucose, 3.2-3.6 g/L citrate. a: Oxygen transfer rate (OTR), b: Final optical density (OD) and pH, c: Final maltose, glucose and citrate concentration, d: Final acetoin and 2,3-butanediol concentration. a: For clarity, only every 10th measuring point over time is marked as a symbol. Mean values for OTR of four replicates with standard deviations as shadows are shown. b-d: For offline analysis, samples (wells) of the replicates of the OTR measurement were pooled at the end of the experiments. OD measurement of pooled samples was performed in triplicate and mean values with standard deviations depicted as error bars are shown. pH and concentrations of sugars and metabolites were determined in a single measurement of pooled samples. c, d: d.l. means that concentrations of components were lower than the detection limit. Final lactate concentration was lower than the detection limit. Parameters in b-d were determined after 63.3 h.

Cultivation conditions: temperature 33 °C, 48-round well plate, filling volume 0.8 mL, shaking frequency 1000 rpm, shaking diameter 3 mm, 0.1 M MES, initial pH 6.5.

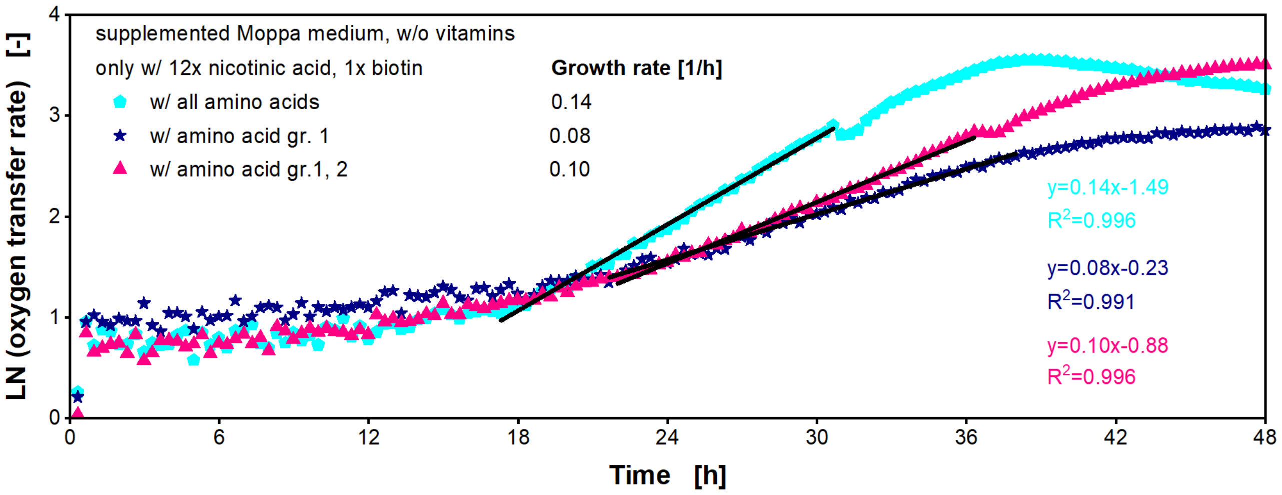

Figure S11: Determination of growth rate for *Paenibacillus polymyxa* cultivation with varying amino acid composition. Supplemented Moppa medium (specified in Table 2) without vitamins (only with nicotinic acid and biotin) and with or without amino acid groups, specified in Additional file 1: Table S2. Growth rates are calculated based on regression of the linear range of logarithm of oxygen transfer rate (OTR). OTRs are shown in Figure 5a. Cultivation conditions: temperature 33 °C, 48-round well plate, filling volume 0.8 mL, shaking frequency 1000 rpm, shaking diameter 3 mm.

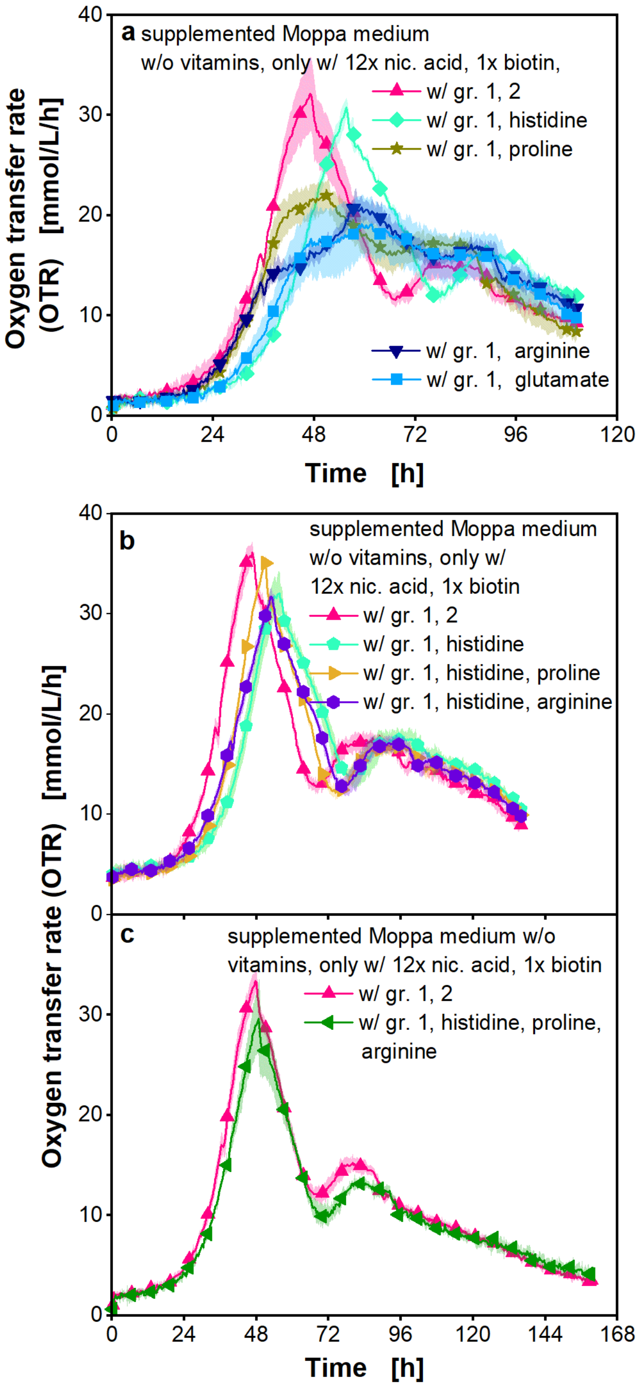

Figure S12: Cultivation of *Paenibacillus polymyxa* with single amino acids of group 2 in microtiter plate. Supplemented Moppa medium (specified in Table 2) without vitamins (only with nicotinic acid and biotin) and without amino acids (only with amino acid group 1) and with or without amino acids of group 2 specified in Additional file 1: Table S2. a, b, c: Oxygen transfer rate (OTR). For clarity, only every 20th measuring point over time is marked as a symbol. Mean values for OTR of at least four replicates with standard deviations as shadows are shown. Cultivation conditions: temperature 33 °C, 48-round well plate, filling volume 0.8 mL, shaking frequency 1000 rpm, shaking diameter 3 mm.

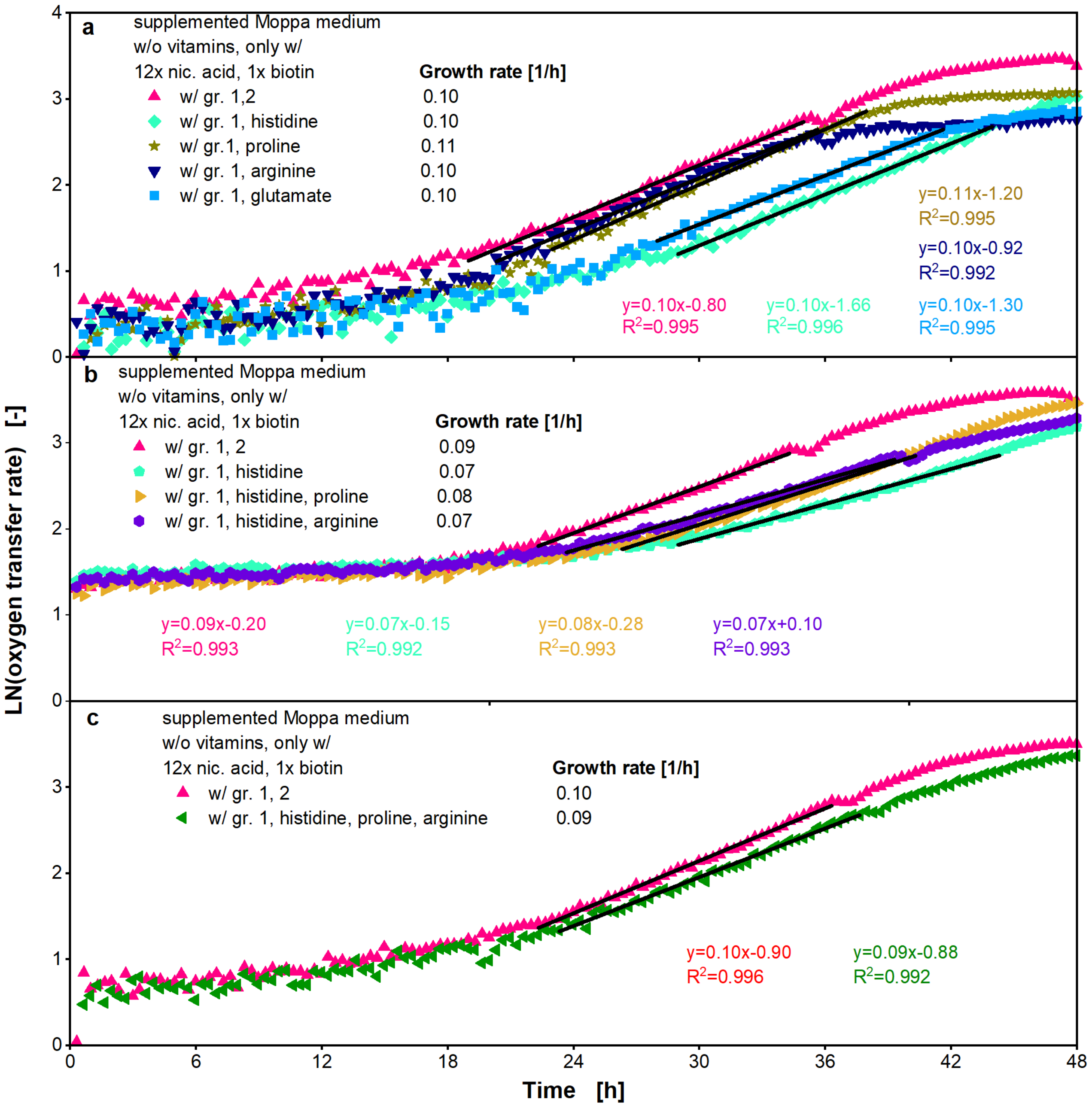

Figure S13: Determination of growth rate for *Paenibacillus polymyxa* cultivation with single amino acids of group 2. Supplemented Moppa medium (specified in Table 2) without vitamins (only with nicotinic acid and biotin) and without amino acids (only with amino acid group 1) and with or without amino acids of group 2 specified in Additional file 1: Table S2. a, b, c: Growth rates are calculated based on regression of the linear range of logarithm of oxygen transfer rate (OTR). OTRs are shown in Additional file 1: Figure S12. Cultivation conditions: temperature 33 °C, 48-round well plate, filling volume 0.8 mL, shaking frequency 1000 rpm, shaking diameter 3 mm.

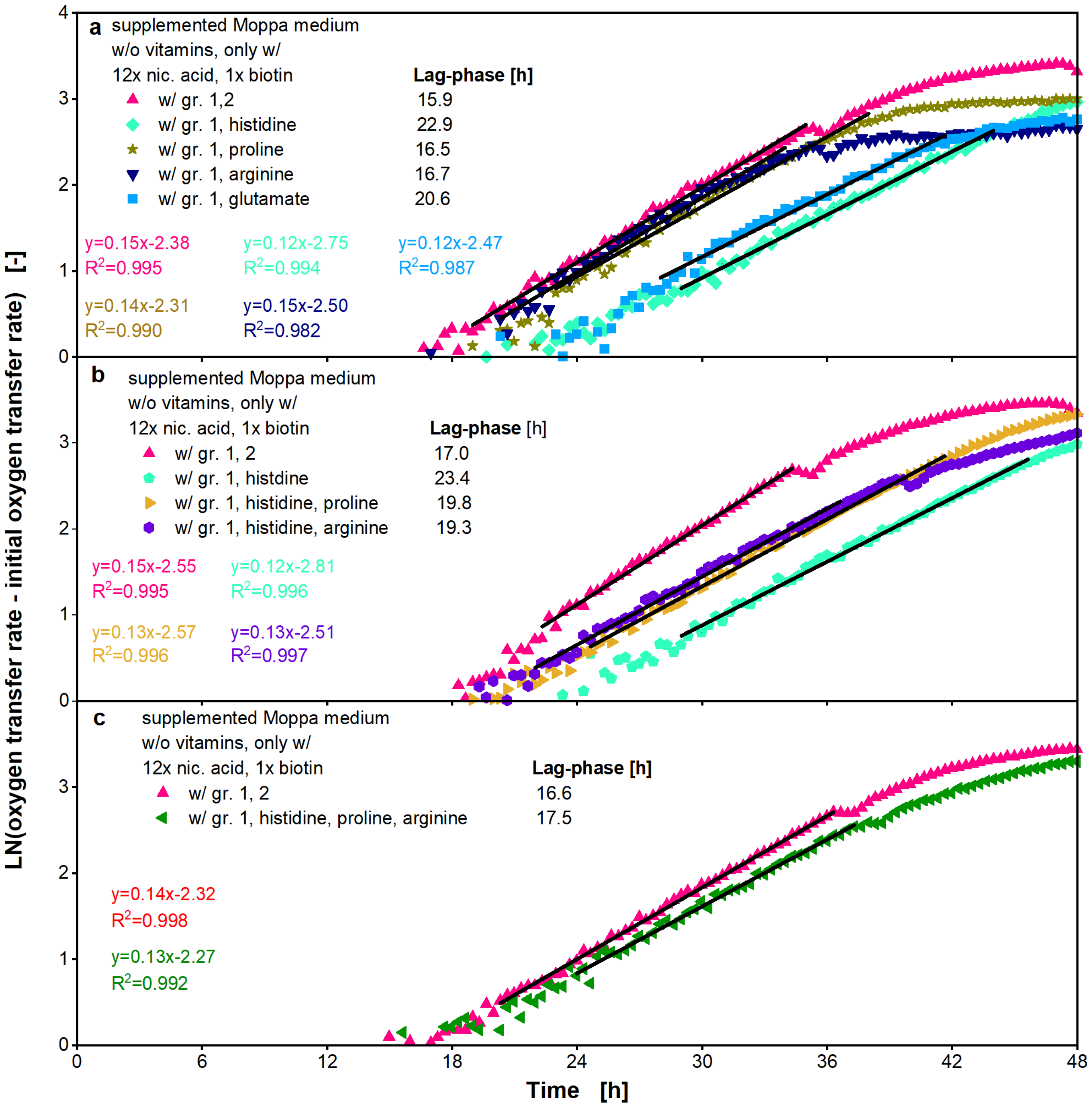

Figure S14: Determination of lag-phase for *Paenibacillus polymyxa* cultivation with single amino acids of group 2. Supplemented Moppa medium (specified in Table 2) without vitamins (only with nicotinic acid and biotin) and without amino acids (only with amino acid group 1) and with or without amino acids of group 2 specified in Additional file 1: Table S2. a, b, c: The logarithm of the difference of the oxygen transfer rate (OTR) and initial OTR was plotted over time. The initial OTR was determined based on the mean value of OTR values between 1.6 and 3.0 h. The lag-phases are calculated based on the intersection point of the regression line of the linear range. OTRs are shown in Additional file 1: Figure S12. Cultivation conditions: temperature 33 °C, 48-round well plate, filling volume 0.8 mL, shaking frequency 1000 rpm, shaking diameter 3 mm.

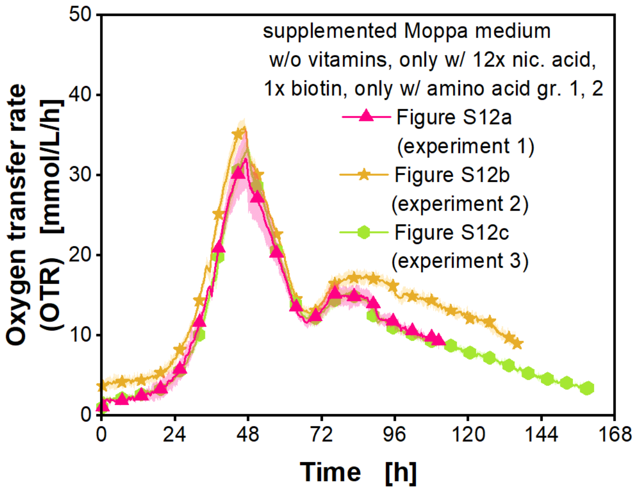

Figure S15: Reproducibility of respiration activity of *Paenibacillus polymyxa* in chemically defined medium in microtiter plate. Supplemented Moppa medium (specified in Table 2) without vitamins (only with nicotinic acid and biotin) and without amino acids (only with amino acid group 1 and 2 specified in Additional file 1: Table S2). Oxygen transfer rate (OTR). For clarity, only every 20th measuring point over time is marked as a symbol. Mean values for OTR of four replicates with standard deviations as shadows are shown. OTRs are also shown in Additional file 1: Figure S12. Cultivation conditions: temperature 33 °C, 48-round well plate, filling volume 0.8 mL, shaking frequency 1000 rpm, shaking diameter 3 mm.

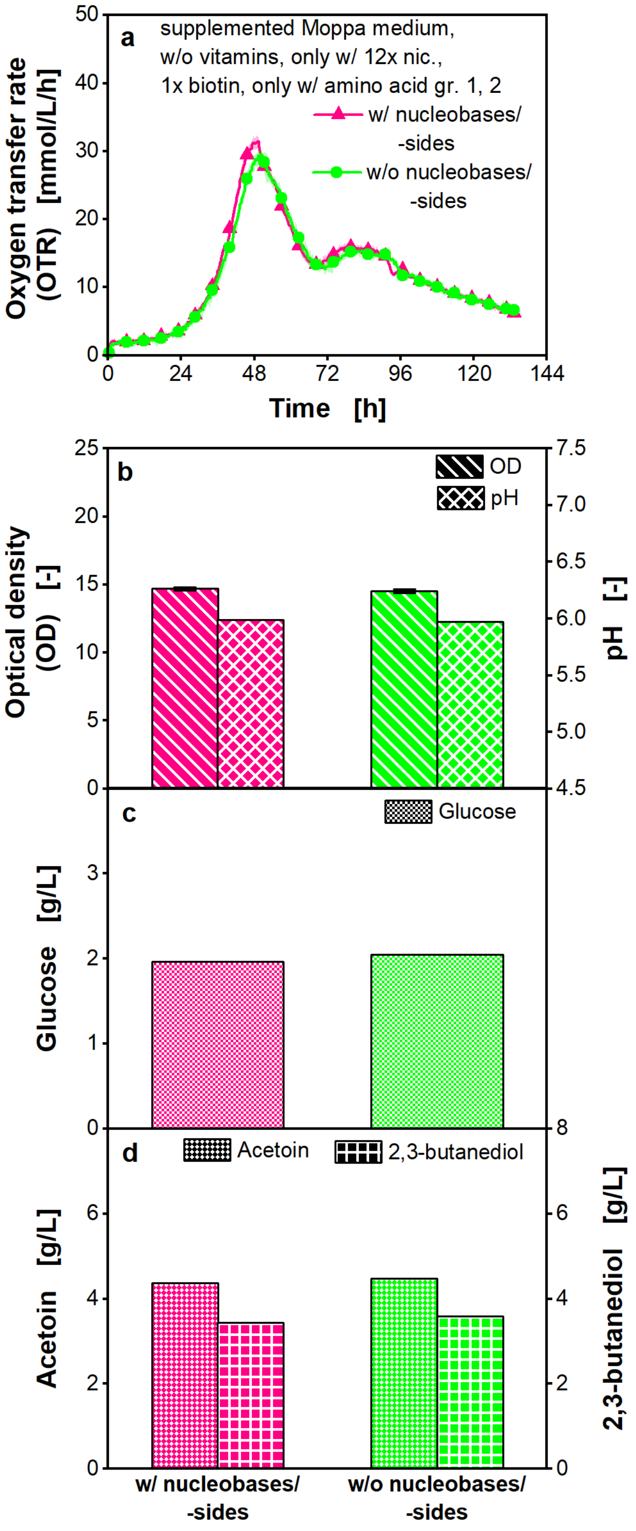

Figure S16: Cultivation of *Paenibacillus polymyxa* without nucleobases/ -sides in microtiter plate. Supplemented Moppa medium (specified in Table 2) without vitamins (only with nicotinic acid and biotin) and without amino acids (only with amino acid group 1 and 2) and with or without nucleobases/-sides. nic.: nicotinic. Amino acids groups (gr.) are specified in Additional file 1: Table S2. Initial concentrations were: 57.4-58.2 g/L maltose, 3.3 g/L glucose, 3.1 g/L citrate. a: Oxygen transfer rate (OTR), b: Final optical density (OD) and pH, c: Final glucose concentration, d: Final acetoin and 2,3-butanediol concentration. a: For clarity,

only every 18th measuring point over time is marked as a symbol. Mean values for OTR of at least four replicates with standard deviations as shadows are shown. Standard deviations are not well recognizable, because they are small. b-d: For offline analysis, samples (wells) of the replicates of the OTR measurement were pooled at the end of the experiments. OD measurement of pooled samples was performed in triplicate and mean values with standard deviation depicted as error bars are shown. pH and concentrations of sugars and metabolites were determined in a single measurement of pooled samples. Final maltose, citrate and lactate concentrations were lower than the detection limit. Parameters in b-d were determined after 133.3 h. Cultivation conditions: temperature 33 °C, 48-round well plate, filling volume 0.8 mL, shaking frequency 1000 rpm, shaking diameter 3 mm.

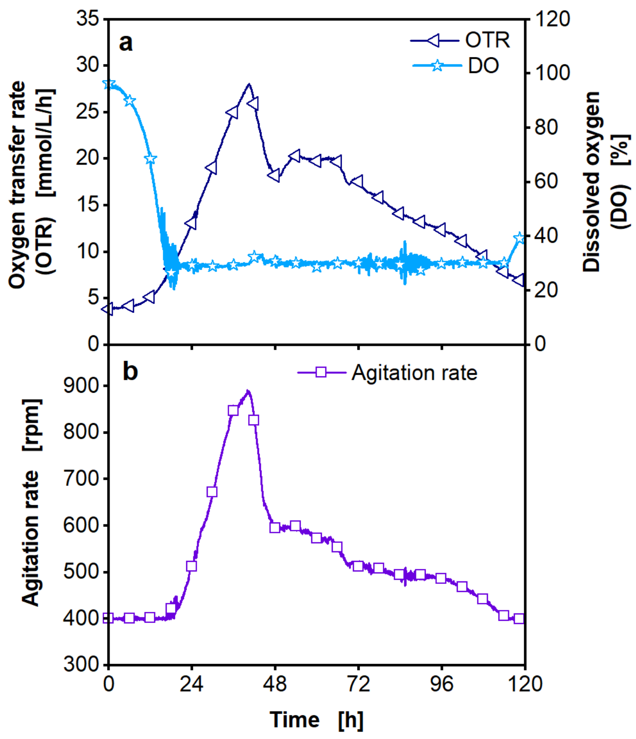

Figure S17: Dissolved oxygen and agitation speed in the cultivation of *Paenibacillus polymyxa* in fermenter. The fermentation is shown in Figure 7. Reduced Moppa medium (specified in Table 2). a: Oxygen transfer rate (OTR) and dissolved oxygen (DO), b: Agitation rate. For clarity, only every 720th measuring point is marked as a symbol for OTR, DO and agitation rate. Cultivation conditions: temperature 33 °C, filling volume 1 L, pH control at pH 6.5, without MES buffer.

References of additional file 1

19. Müller J, Beckers M, Mußmann N, Bongaerts J, Büchs J. Elucidation of auxotrophic deficiencies of *Bacillus pumilus* DSM 18097 to develop a defined minimal medium. Microb Cell Fact. 2018;17:106. doi:10.1186/s12934-018-0956-1.

30. Poolman B, Konings WN. Relation of growth of *Streptococcus lactis* and *Streptococcus cremoris* to amino acid transport. J Bacteriol. 1988;170:700–7. doi:10.1128/jb.170.2.700-707.1988.

31. Wilming A, Begemann J, Kuhne S, Regestein L, Bongaerts J, Evers S, et al. Metabolic studies of γ-polyglutamic acid production in *Bacillus licheniformis* by small-scale continuous cultivations. Biochem Eng J. 2013;73:29–37. doi:10.1016/j.bej.2013.01.008.

51. Adlakha N, Pfau T, Ebenhöh O, Yazdani SS. Insight into metabolic pathways of the potential biofuel producer, *Paenibacillus polymyxa* ICGEB2008. Biotechnol Biofuels. 2015;8:159. doi:10.1186/s13068-015-0338-4.

57. Mühlmann M, Forsten E, Noack S, Büchs J. Optimizing recombinant protein expression via automated induction profiling in microtiter plates at different temperatures. Microb Cell Fact. 2017;16:220. doi:10.1186/s12934-017-0832-4.
